# C_4_ trees have a broader niche than their close C_3_ relatives

**DOI:** 10.1093/jxb/erac113

**Published:** 2022-05-23

**Authors:** Sophie N R Young, Luke T Dunning, Hui Liu, Carly J Stevens, Marjorie R Lundgren

**Affiliations:** 1 Lancaster Environment Centre, Lancaster University, Lancaster LA1 4YQ, UK; 2 Ecology and Evolutionary Biology, School of Biosciences, University of Sheffield, Sheffield S10 2TN, UK; 3 Key Laboratory of Vegetation Restoration and Management of Degraded Ecosystems, Guangdong Provincial Key Laboratory of Applied Botany, South China Botanical Garden, Chinese Academy of Sciences, Xingke Road 723, Guangzhou 510650, China

**Keywords:** Biogeography, C_4_ photosynthesis, *Chamaesyce*, *Euphorbia*, euphorbiaceae, trees

## Abstract

Previous studies have demonstrated the ecological sorting of herbaceous C_3_ and C_4_ species along gradients of precipitation and temperature: C_4_ herbaceous species typically occupy drier and warmer environments than their C_3_ relatives. However, it is unclear if this pattern holds true for C_4_ tree species, which are unique to Euphorbiaceae and found only on the Hawaiian Islands. Here, we combine occurrence data with local environmental and soil datasets to, for the first time, distinguish the ecological factors associated with photosynthetic diversification in the tree life form. These data are presented within a phylogenetic framework. We show that C_3_ and C_4_ trees inhabit similar environments, but that C_4_ photosynthesis expands the ecological niche in trees relative to that of C_3_ tree species. In particular, when compared with C_3_ trees, C_4_ trees moved into higher elevation habitats with characteristically sparse vegetation (and thus greater sunlight) and cooler temperatures, a pattern which contrasts with that of herbaceous species. Understanding the relationship between C_4_ photosynthesis and ecological niche in tree species has implications for establishing how C_4_ photosynthesis has, in this rare instance, evolved in trees, and whether this unique combination of traits could be exploited from an engineering perspective.

## Teaser

While C_3_and C_4_trees inhabit similar environments, C_4_photosynthesis in trees expands the ecological niche relative to that of C_3_tree species.

## Introduction

It is widely accepted that the ecological niche of a given plant species is influenced by its photosynthetic efficiency (Black, 1971; Sage *et al.*, 1999; Lundgren *et al.*, 2015). Modifications to the photosynthetic apparatus can increase or decrease efficiency depending on environmental conditions. One such modification that increases carbon, water, and nitrogen use efficiencies is the C_4_ photosynthetic pathway (Evans, 2013). This pathway largely eliminates the energetically costly process of photorespiration, which occurs when the carbon-fixing enzyme Rubisco catalyses the fixation of oxygen instead of CO_2_, resulting in the accumulation of toxic by-products that need to be recycled (Bräutigam and Gowik, 2016). Certain environmental conditions are known to increase the rate of photorespiration, including low CO_2_ concentrations, warmth, bright light, aridity, and salinity (Chollet and Ogren, 1975; Ehleringer *et al.*, 1991; Sage *et al.*, 2018). As a result, species utilizing C_4_ photosynthesis theoretically perform better than plants using the ancestral C_3_ pathway in these environments (Pearcy and Ehleringer, 1984). This, at least in part, explains the well-reported differences in global distribution patterns for C_3_ and C_4_ species (reviewed in Ehleringer *et al.*, 1997; Christin and Osborne, 2014).

Ecological sorting of photosynthetic types along temperature gradients is particularly apparent in grasses, where C_4_ grasses are abundant at high temperatures and give way to C_3_ grasses as temperature declines (Teeri and Stowe, 1976; Bremond *et al.*, 2012; Pau *et al.*, 2013). For eudicot herbaceous species, water availability seems to be the most important determinant of distribution, with C_4_ forbs being favoured over their C_3_ counterparts in areas with limited water supply (Stowe and Teeri, 1978; Pyankov *et al.*, 2010). Furthermore, species using the CAM (Crassulacean acid metabolism) photosynthetic pathway have advantages over both C_3_ and C_4_ species with respect to their maximum potential water use efficiencies, and frequently dominate in the most arid environments (Orsenigo *et al.*, 1997; Black and Osmond, 2003). However, epiphytic CAM species can also be abundant in tropical rainforests, which have very high levels of precipitation, as it is the epiphytic growth form which drives their water limitation (Whittaker, 1975; Quezada and Gianoli, 2011). The difference in the major environmental predictors of photosynthetic type distribution between C_3_ and C_4_ grasses and forbs, as well as the contrasting environments of CAM species highlights the importance of distinguishing growth forms when examining the effect of photosynthetic type on the ecological niche. However, the relationship between photosynthetic type and ecology has not yet been investigated in the tree growth form.

The effects of photosynthetic type on the ecological and geographical distributions of trees may be different from those of herbaceous species for three reasons. First, there are key differences between monocots and eudicots, the plant clades which contain all known C_4_ species (Sage, 2016). More than half of all the >60 C_4_ origins have occurred in eudicots, but these lineages account for less than a quarter of all known C_4_ species (Sage, 2016). Quantum yield (defined as the rate of photosynthesis relative to photon absorption) is generally lower in the eudicots—which includes almost all true tree species—than in the monocots. This results in generally poorer shade tolerance among C_4_ eudicots compared with monocots that could have implications for their distributions, and thus the relationship between photosynthetic type and ecological niche (Ehleringer *et al.*, 1997). This poor shade tolerance may be augmented by a potentially reduced capacity for exploiting sunflecks in C_4_ versus C_3_ plants, although this response does not appear to be consistent or universal given that reduced sunfleck use efficiency is not observed in all species and growth forms of C_4_ plants (Pearcy *et al.*, 1985; Krall and Pearcy, 1993; discussed in Sage and McKown, 2006; Sage, 2014). Secondly, life history influences ecological niche (Pyankov *et al.*, 2010; Liu *et al.*, 2019). Pyankov *et al.* (2010) hypothesize that the abundance of C_4_ annual species compared with C_4_ perennials reflects the fact that C_4_ photosynthesis confers the greatest fitness benefit over short periods of time. Therefore, tree species, which have long life spans, may not benefit as much from C_4_ photosynthesis as short-lived herbaceous species. Furthermore, Liu *et al.* (2019) demonstrate that the variation in niche descriptors of C_3_ and C_4_ subtropical grasses was best explained by differences in life history: annual subtropical grasses (particularly C_4_ annuals) tend to grow in regions with higher temperatures and lower, more seasonal precipitation compared with perennial grasses. Thirdly, growth form probably influences the ecological niche through functions such as water transport or light competition rather than photosynthetic type. Tall plant species such as trees typically exhibit wider water conduits than shorter plants, and these wider conduits are more vulnerable to embolism under drought or freezing (Olson *et al.*, 2018). This means that the tree state may result in an intrinsically higher degree of vulnerability to drought, and this vulnerability would not necessarily be alleviated by the C_4_ pathway. This problem may be exacerbated where the C_4_ state is recently evolved (i.e. a tree evolves C_4_ or the tree state evolves in a young C_4_ lineage), as shown by a study of young C_4_ grass lineages which demonstrates that leaf hydraulic conductance is increased due to the anatomy required by the C_4_ system, despite the C_4_ state reducing hydraulic demand, resulting in less negative turgor loss points in young compared with old C_4_ lineages (Zhou *et al.*, 2020, Preprint). As such, a tall C_4_ plant such as a tree, particularly one belonging to a young C_4_ lineage, would theoretically not be able to access more arid environments than their C_3_ counterparts. This is reflected in the apparent height limitation of the only known C_4_ trees (found in one of the older C_4_ eudicot lineages), which are not observed to exceed 10 m in height (Young *et al.*, 2020). Despite these clear differences across life forms, histories, and growth patterning, the ecological sorting of photosynthetic diversity in trees remains largely unexplored.

Evolutionary history is another important factor determining the ecological sorting of photosynthetic diversity. Comparisons between distantly related grass species have often revealed more pronounced differences between the ecological distributions of C_3_ and C_4_ species than studies comparing photosynthetic diversity between closely related species or within a single species (Pau *et al.*, 2013; Lundgren *et al.*, 2015). The broad differences identified between C_3_ and C_4_ species probably reflect the fact that distribution patterns are not solely determined by the acquisition of C_4_ photosynthesis and are affected by the ecology and functional traits of the ancestral C_3_ lineages (Edwards and Still, 2008; Edwards and Smith, 2010). With time, niche specialization may then occur after the initial emergence of C_4_ physiology, depending on differences in functional traits, resulting in some C_4_ taxa becoming specialized to environments different from those of their C_3_ ancestors and generating an apparent niche shift. This means that comparisons between distantly related species of differing photosynthetic type may reveal pronounced differences between C_4_ and C_3_ species, but these differences may only be partly driven by photosynthetic type. Furthermore, studies that look at intraspecific photosynthetic diversity offer a closer look at the direct consequences of C_4_ physiology. Indeed, a study on the intraspecific photosynthetic diversity of the grass *Alloteropsis semialata*—the only species with both C_4_ and non-C_4_ genotypes—showed that C_4_ photosynthesis actually broadens the ecological niche, rather than shifting it away from that of ancestral C_3_ lineages (Lundgren *et al.*, 2015). With this in mind, our study set out to determine the ecological sorting of closely related C_3_ and C_4_ trees, as well as any influence that evolutionary history may have had on their ecological distributions.

Tree photosynthetic diversity only exists within Euphorbiaceae, a morphologically diverse plant family with C_3_, C_4_, and CAM tree species (Table 1; Webster *et al.*, 1975; Webster, 1994; Horn *et al.*, 2012). The Chamaesyce clade of *Euphorbia* (Euphorbiaceae) is the largest single C_4_ lineage among the eudicots, with 350 C_4_ species and containing the only true C_4_ trees, defined here as tall, perennial, woody life forms with secondary growth and C_4_ leaves (Pearcy and Troughton, 1975; Yang and Berry, 2011; Young *et al.*, 2020). These C_4_ trees are endemic to the Hawaiian Islands, where they diversified from a likely herbaceous ancestor that arrived on the islands ~5 million years ago (Yang *et al.*, 2018). This diversification event yielded five C_4_ trees in present-day Hawaii, which, when combined with the 17 C_3_ and three CAM Euphorbiaceae tree species currently on these islands (Table 1), makes the Hawaiian Islands the global centre of photosynthetic diversity in trees. The environment on the islands is highly heterogenous, and trees in Euphorbiaceae occupy a range of environments from bright, open scrubland to mesic forest where they experience differing temperatures, precipitation levels, and light availability (Table 1; Sporck, 2011; Sage and Sultmanis, 2016). Hawaiian trees in Euphorbiaceae therefore provide a unique opportunity to compare the ecological niches of tree species with diverse photosynthetic backgrounds from a similar geographic region.

**Table 1.** Number of occurrences (*n*), photosynthetic type (C_3_, C_2_, C_4_, or CAM), and life form of species in Euphorbiaceae occurring on the Hawaiian Islands

Species	*n*	Photosynthetic type	δ ^13^C a	Reference	Life form (WCSP ^*b*^)	Environmental data from herbarium record ^*c*^
**Tree**						
*Euphorbia atrococca*	6	C_4_	–12.40	Pearcy and Troughton (1975)	phan	Disturbed lowland forest
*Euphorbia celastroides*	43	C_4_	–13.21	Pearcy and Troughton (1975), Horn *et al.* (2014)	phan	Highly variable: grassy or rocky windswept slopes to open forest to bog
*Euphorbia herbstii*	4	C_4_	–12.80	Pearcy and Troughton (1975) ^*d*^	phan	Forested slopes, deep shade
*Euphorbia olowaluana*	12	C_4_	–14.40	Pearcy and Troughton (1975)	phan	Dry forest, lava field
*Euphorbia rockii*	4	C_4_	–12.95	Pearcy and Troughton (1975)	phan	Open bog, open rainforest on hilltop, moist rainforest, exposed ridge, wooded stream bank, high elevations
*Acalypha wilkesiana*	7	C_3_	NA		nanophan/phan	
*Aleurites moluccanus*	107	C_3_	NA		phan	
*Claoxylon sandwicense*	18	C_3_	NA	Robichaux and Pearcy (1980)	nanophan/phan	Wooded slope, dry forest
*Cnidoscolus aconitifolius*	1	C_3_	NA		nanophan/phan	
*Croton guatemalensis*	1	C_3_	NA		phan	
*Euphorbia cotinifolia*	1	C_3_	–23.93	Horn *et al.* (2014)	nanophan/phan	
*Euphorbia haeleeleana*	3	C_3_	NA	Morden *et al.* (2014) ^*e*^	phan	Mesic forest
*Euphorbia leucocephala*	2	C_3_	–27.58	Horn *et al.* (2014)	nanophan/phan	
*Hevea brasiliensis*	1	C_3_	–28.90	Martinelli *et al.* (1998)	phan	
*Homalanthus populifolius*	3	C_3_	NA		nanophan/phan	
*Hura crepitans*	1	C_3_	–26.67	Horn *et al.* (2014)	phan	
*Jatropha integerrima*	16	C_3_	NA		nanophan/phan	
*Jatropha multifida*	2	C_3_	NA		nanophan/phan	
*Macaranga mappa*	18	C_3_	NA		phan	
*Macaranga tanarius*	21	C_3_	NA		phan	
*Manihot carthaginensis*	5	C_3_	NA		nanophan/phan	
*Vernicia fordii*	1	C_3_	NA		phan	
*Euphorbia lactea*	4	CAM	–14.79	Horn *et al.* (2014), Mason *et al.* (2015)	succ nanophan/phan	
*Euphorbia tirucalli*	10	CAM/C_3_	–16.45	Horn *et al.* (2014), Bender (1971), Bender *et al.* (1973)	succ nanophan/phan	
*Jatropha curcas*	3	CAM/C_3_	NA	Winter and Holtum (2015)	nanophan/phan	
**Shrub**						
*Euphorbia arnottiana*	2	C_4_	–13.40	Pearcy and Troughton (1975), Yang *et al.*, 2018	cham	
*Euphorbia atoto*	1	C_4_	–12.4	Pearcy and Troughton (1975)	cham/nanophan	
*Euphorbia clusiifolia*	2	C_4_	–12.20	Pearcy and Troughton (1975)	–	
*Euphorbia degeneri*	7	C_4_	–12.9	Pearcy and Troughton (1975)	–	
*Euphorbia eleanoriae*	1	C_4_	NA	Yang *et al.* (2018)	–	
*Euphorbia halemanui*	6	C_4_	NA	Yang *et al.* (2018)	–	
*Euphorbia kuwaleana*	1	C_4_	NA	Yang *et al.* (2018)	phan	Grassy hilltop, volcanic rock
*Euphorbia multiformis*	13	C_4_	–12.60	Pearcy and Troughton (1975), Yang *et al.* (2018)	–	
*Euphorbia remyi*	7	C_4_	–13.10	Pearcy and Troughton (1975)	–	
*Euphorbia skottsbergii*	7	C_4_	–12.00	Pearcy and Troughton (1975)	–	
*Euphorbia sparsiflora*	2	C_4_	NA	Yang *et al.* (2018)	–	
*Acalypha hispida*	6	C_3_	NA		nanophan	
*Codiaeum variegatum*	23	C_3_	NA		nanophan/phan	
*Jatropha podagrica*	11	C_3_	NA		nanophan	
*Manihot esculenta*	4	C_3_	NA	De Souza *et al.* (2017) f	nanophan/phan	
*Ricinus communis*	82	C_3_	–30.2	Bender (1971)	nanophan/phan	Side of valley
*Euphorbia milii*	2	CAM/C_2_	–21.70	Horn *et al.* (2014), Herrera (2013)	cham/nanophan	
*Euphorbia tithymaloides*	2	CAM/C_3_	–23.94	Horn *et al.* (2014), Ramachandra et al. (2003)	nanophan	
**Herb**						
*Euphorbia hirta*	71	C_4_	–13.08	Horn *et al.* (2014), Yang and Berry (2011)	ther	
*Euphorbia hypericifolia*	48	C_4_	NA	Yang and Berry (2011)	ther/cham	
*Euphorbia hyssopifolia*	9	C_4_	NA	Yang and Berry (2011)	ther	
*Euphorbia ophthalmica*	3	C_4_	NA	Yang and Berry (2011)	ther	
*Euphorbia prostrata*	38	C_4_	NA	Yang and Berry (2011)	ther	
*Euphorbia serpens*	3	C_4_	NA	Yang and Berry (2011)	ther	
*Euphorbia thymifolia*	22	C_4_	NA	Yang and Berry (2011)	ther/hel	
*Euphorbia heterophylla*	22	C_3_	–29.67	Horn *et al.* (2014), Ramachandra and Das (1986)	ther	
*Euphorbia peplus*	1	C_3_	–33.32	Horn *et al.* (2014)	ther	

^*a*^ δ^13^C data (averaged where multiple measurements were found) are included where available in the literature, NA = not available.

^*b*^ Life form is obtained from the Kew World Checklist of Selected Plant Families (WCSP) and species designated as one or a combination of phanerophyte (phan, small and large trees), nanophanerophyte (nanophan, shrub), succulent (succ), chamaephyte (cham, woody or herbaceous perennial), therophyte (ther, annual), or helophyte (hel, herbaceous species with roots in water/saturated soil). These life form designations were used in conjunction with information from the citations to group species into trees, shrubs, and herbs.

^*c*^ Environmental data for selected tree and shrub species were recorded from herbarium datasheets available online at GBIF.

^*d*^ Named here as *E. forbesii*.

^*e*^ Secondary source naming *E. haeleeleana* as C_3_.

^*f*^ Low level CAM, possibly shifts to CAM under drought.

Here, we combine geographical occurrence and environmental datasets in a phylogenetic framework to examine the phylogeography of photosynthetic diversity in trees in order to elucidate the geographical and environmental factors that are permissive for photosynthetic innovation in trees. Based on our current knowledge of the sorting of photosynthetic diversity among herbaceous species, we hypothesize that C_4_ trees occur in drier, hotter, and more open environments than their C_3_ counterparts (Table 2), with the differences in water availability acting as the most important determinant of distribution as for herbaceous eudicots. However, as discussed above, these environmental differences may be less pronounced than those for herbaceous species given the theoretically reduced benefits of C_4_ photosynthesis over long life spans and in the tree growth form. With only three CAM tree species which occur in small numbers on the Hawaiian Islands, we were unable to perform robust analyses on the phylogeography of this photosynthetic type but instead report on its distribution in relation to C_3_ and C_4_ trees in our study.

**Table 2.** Environmental parameters hypothesized to differ by photosynthetic type in trees

Type of parameter	Parameter	Hypothesis	Supporting reference ^*a*^
Climatic	Minimum Temperature of Coldest Month	C_4_ trees are found in areas with lower minimum monthly temperatures than C_3_ trees	Edwards and Smith (2010)
	Temperature of the Wettest Quarter (Growing Season)	C_4_ trees are found in areas with higher growing season temperatures than C_3_ trees	Teeri and Stowe (1976), Vogel *et al.* (1986)
	Precipitation of Driest Month	C_4_ trees are found in areas with lower minimum monthly precipitation levels than C_3_ trees	Pyankov *et al.* (2010)
	Precipitation Seasonality	C_4_ trees are found in areas with more seasonal precipitation than C_3_ trees	Edwards and Smith (2010)
	Solar Radiation	C_4_ trees are found in areas with higher solar radiation than C_3_ trees	Ehleringer (1978)
	Climatic Water Deficit	C_4_ trees are found in areas with higher climatic water deficits than C_3_ trees	Witwickiet al. (2016)
Ecological	Vegetation Cover	C_4_ trees are found in areas with lower vegetation cover than C_3_ trees	Pau *et al.* (2013), Still *et al.* (2014)

^*a*^ Supporting references are studies showing environmental distributions for C_3_ and C_4_ herbaceous species.

## Materials and methods

### Geographic distribution data for Euphorbiaceae species

Occurrence data for all species in Euphorbiaceae on the Hawaiian Islands were downloaded from the Global Biodiversity Information Facility (https://www.gbif.org, https://doi.org/10.15468/dl.bc6bus) and the Botanical Information and Ecology Network (BIEN; https://bien.nceas.ucsb.edu/bien/). Data were imported into R using the readr and BIEN packages in R, respectively (Maitner *et al.*, 2018; Wickham *et al.*, 2018; R Core Team, 2020). These combined datasets yielded 1509 occurrence records for 78 species.

Occurrence data were then cleaned in R to remove any records that potentially contained inaccurate or unreliable information. First, all latitude and longitude data were rounded to two decimal places, and records with incomplete coordinate, species, or country data were removed. Taxa names were updated to currently accepted names where appropriate. Secondly, any records that were identified by GBIF as having at least one of the issues listed in Supplementary Table S1 were removed. Thirdly, data were processed using the CoordinateCleaner package in R to flag records with coordinates corresponding to country capitals or centroids, the GBIF headquarters, or another biodiversity institution; records with equal, zero, or invalid values for latitude and longitude; and records with coordinates that were not on land (Zizka *et al.*, 2019). A total of 277 flagged records (summarized in Supplementary Fig. S1) were removed. Finally, duplicate records and records associated with botanic gardens, private gardens, and arboretums were removed. Upon completion of these cleaning steps, 690 occurrence records for 52 species remained (Supplementary Fig. S2).

The remaining records were then designated as herb, shrub, or tree based on life form information in the World Checklist of Selected Plant Families (WCSP, 2021; Table 1). For Hawaiian *Euphorbia*, Yang *et al.* (2018) was used to supplement the life form data from the WCSP. Occurrence records were also designated as having C_3_, C_4_, C_2_, or CAM photosynthetic type based on the information available in the literature (Table 1; Supplementary Fig. S3). All C_4_ Hawaiian species were identified using Yang *et al.* (2018) and Yang and Berry (2011). Five CAM species were identified to occur on the Hawaiian Islands, including three CAM tree species, *Jatropha curcas*, *Euphorbia lactea*, and *Euphorbia tirucalli* (Mies *et al.*, 1996; Mason *et al.*, 2015; Winter and Holtum, 2015). All other records, including those whose photosynthetic type could not be found in the literature, were designated as C_3_ (Table 1). Where available from the literature, δ^13^C stable isotope values are recorded in Table 1 to support these photosynthetic type designations, with values greater (i.e. less negative) than –15 per mille indicating likely C_4_ biochemistry.

### Environmental factors

Biogeographical parameters hypothesized to be associated with the sorting of photosynthetic types were selected for our analyses, including geographical, climatic, and ecological factors (Table 2). Elevation data for occurrence records in Hawaii were obtained from the Shuttle Radar Topography Mission (STRM) digital elevation model (DEM) at 1 arc-second (~30 m) spatial resolution via the rgbif package in R (Farr *et al.*, 2007; Chamberlain *et al.*, 2022). Environmental and ecological data were obtained at ~250 m resolution from the Online Rainfall Atlas of Hawaii and Climate of Hawaii (Giambelluca *et al.*, 2013, 2014). Monthly precipitation and temperature data were processed using the bioclim2 function from the climates package in R to recreate the standard 19 bioclimatic variables (VanDerWal *et al.*, 2014). Climatic water deficit (CWD) was calculated as potential evapotranspiration minus actual evapotranspiration (Stephenson, 1998). C_3_ and C_4_ tree data were plotted onto the Whittaker biomes using the plotbiomes package in R (Stefan and Levin, 2019). Soil data for the Hawaiian Islands were obtained from the Hawaii Soil Atlas (http://gis.ctahr.hawaii.edu/SoilAtlas) at ~670 m spatial resolution and imported into ArcGIS Pro (https://www.esri.com/en-us/arcgis/products/arcgis-pro/overview). Data were obtained for the following soil variables: fertility (including soil class and cation exchange capacity), order, organic matter content, pH, shrink–swell potential (soil stability), and water permeability. Soil data for coordinates of interest were then extracted via the Spatial Join function from the Analysis toolbox.

### Phylogeny

A dated phylogenetic tree for Euphorbiaceae was generated using previously published data. Three plastid regions (*trnK–matK*, *rbcL*, and *trnL–trnF*) were targeted as they were the best represented for the required taxa. Initially, the *trnK–matK* and *rbcL* dataset of Tokuoka (2007) was used, adding additional taxa and *trnL–trnF* sequence information where available. Each gene was aligned with MAFFT v.7.017 (Katoh *et al.*, 2002) and concatenated into a single alignment. The final alignment was 4507 bp long and included 112 species (see Supplementary Table S2 for a summary of sequence data used and accession numbers; see Supplementary Fig. S4 for a phylogenetic tree with all 112 species). A dated phylogeny was inferred using BEAST2 v2.6.3 (Bouckaert *et al.*, 2014) through the Cyberinfrastructure for Phylogenetic Research (CIPRES) Science Gateway V.3.3. To date the tree, a secondary calibration point from Horn *et al.* (2014) was used, fixing the divergence time between *Euphorbia herbstii* and *Hura crepitans* at 64.6076 million years ago (SD=0.0001). Three different analyses were run with 100 000 000 generations, sampling a tree every 1000 generations, a Yule speciation process, a relaxed log-normal clock, and the GTR+G model. The convergence of all three runs was verified using Tracer v. 1.6.0 (Drummond and Rambaut, 2007), after which a burn-in period of 10% was set. All trees were concatenated, and a maximum credibility tree inferred mapping median ages onto the nodes.

### Statistical analyses

Statistical analyses were conducted in R version 4.0.4 (R Core Team, 2020). CAM species were excluded from the analyses owing to the small sample size (0 herb, 4 shrub, and 15 tree occurrence data points). Analyses were conducted primarily on data for C_3_ and C_4_ trees. Data for all C_3_ and C_4_ life forms, including herb and shrub occurrence data, were analysed separately from the tree data (Supplementary Figs S5, S6). Soil data and environmental data were analysed separately from each other due to soil data only being available for a subset (61%) of the occurrence data points (Supplementary Fig. S7).

To explain any ecological variation between trees of C_3_ and C_4_ photosynthetic types, principal component analyses (PCAs) were conducted using the FactoMineR package (Le *et al.*, 2008). Predictor variables that were hypothesized to influence the sorting of photosynthetic types were chosen for the analysis (Table 2), including minimum temperature of the coldest month, temperature of the wettest quarter (proxy for growing season temperature), precipitation seasonality, precipitation of the driest month, CWD, solar radiation, and vegetation cover (i.e. to indicate shaded habitats). Leave-one-out cross-validation was used to estimate the number of dimensions for the PCAs. All variables were scaled to unit variance before fitting. The first PCA was performed on 268 occurrence points representing 22 tree species, comprising 17 C_3_ species (*n*=202) and five C_4_ species (*n*=66). The 95% confidence ellipses were calculated for each group.

A second PCA was conducted on environmental data for C_3_ and C_4_ herbaceous (*n*=21, *n*=178, respectively), shrub (*n*=119, *n*=47), and tree (*n*=202, *n*=66) occurrence points to examine the ecological differences between C_3_ and C_4_ individuals of all life forms (Supplementary Fig. S5). Data for woody species (i.e. trees and shrubs) were considered together in order to examine the ecological differences between woody and non-woody species. The continuous soil variables from the soil dataset were included in a third PCA. These included cation exchange capacity (CEC), organic matter content (OM), pH, shrink–swell potential (soil stability), and water permeability (*K*_sat_). Soil data for C_3_ and C_4_ tree (*n*=134, *n*=29) occurrence points were included in this analysis (Supplementary Fig. S7). In all cases, all variables were scaled to unit variance before fitting.

To identify potential correlation between environmental and geographical distances (i.e. whether C_3_ and C_4_ trees have expanded their environmental range as they have expanded their geographical range across the Hawaiian Islands), Mantel permutation tests were conducted using the ape package (Paradis and Schliep, 2019). Geographic distances between points were extracted and assembled into a matrix using the earth.dist function in the fossil package (Matthew, 2011), and then compared with environmental distances (Euclidian distance in the space formed by the first three axes of the PCA) to test for statistical associations between matrices of environmental and geographical data. Linear regression analysis was carried out by fitting a linear model to each of the C_3_ and C_4_ datasets, with the slope of this regression representing the environmental change per unit of geographical expansion.

To distinguish the role that evolutionary history may have played in photosynthetic type sorting across these six environmental variables, phylogenetic generalized least squares (PGLS) analyses were performed using the pgls function in the caper package in R (Orme *et al.*, 2018). A comparative dataset was assembled from species mean data for each of the seven environmental variables used in the PCA, the first three principal components (PCs) of the PCA, and the species phylogeny. This dataset included a variance–covariance (VCV) matrix that represents species’ phylogenetic relationships to one another. A PGLS model was then fitted to each of the seven environmental variables and three PCs in the dataset with photosynthetic type as the categorical predictor. Pagel’s lambda (λ) was calculated to estimate the strength of the phylogenetic signal in the mean response of each species on a scale of zero (no phylogenetic dependence) to one (perfect phylogenetic dependence). ANOVA was then performed on each model.

## Results

### Geographical and environmental distributions of C_3_ and C_4_ trees overlap

**Fig. 1.**
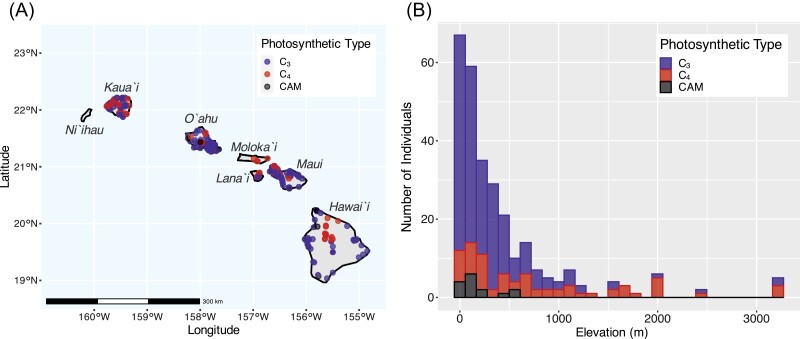
Geographical and topographical distribution of photosynthetic diversity in trees in Euphorbiaceae across the Hawaiian Islands. (A) Individual occurrence points and (B) a histogram of elevations are shown for C_3_ (blue, *n*=202), C_4_ (red, *n*=66), and CAM (black, *n*=15) trees, representing 17, 5, and 3 species, respectively.

C_3_, C_4_, and CAM Euphorbiaceae trees have largely overlapping geographical distributions across the Hawaiian Islands (Fig. 1A). Based on occurrence data available in the BIEN and GBIF databases, both C_3_ and C_4_ trees in Euphorbiaceae occur on the islands of Kaua`i, O`ahu, Lana`i, Maui, and Hawai`i. Only a single Euphorb tree species, *Euphorbia celastroides*, occurs on Molokaa`i. In fact, *E. celastroides* is the only C_4_ tree species recorded on each of Molokaa`i, Lana`i, and Maui, and has the most widespread distribution of any of the C_4_ trees. Only one island—Ni`ihau—has no record of Euphorb trees. There are also three CAM tree species in Euphorbiaceae on the Hawaiian Islands (*Jatropha curcas*, *Euphorbia lactea*, and *E. tirucalli*), which occur on Maui, O`ahu, and Hawai`i (Fig. 1A). Trees in Euphorbiaceae occur across a broad range of elevations across the Islands, from –5 m to 3212 m (Fig. 1B). C_3_ trees occupy this entire range of elevations, but are largely skewed toward the lower elevations, with a median value of 170 m. C_4_ trees are found at elevations of 6 m to 3212 m, and are more evenly distributed across this range than their C_3_ counterparts, with a median elevation of 526 m.

**Fig. 2.**
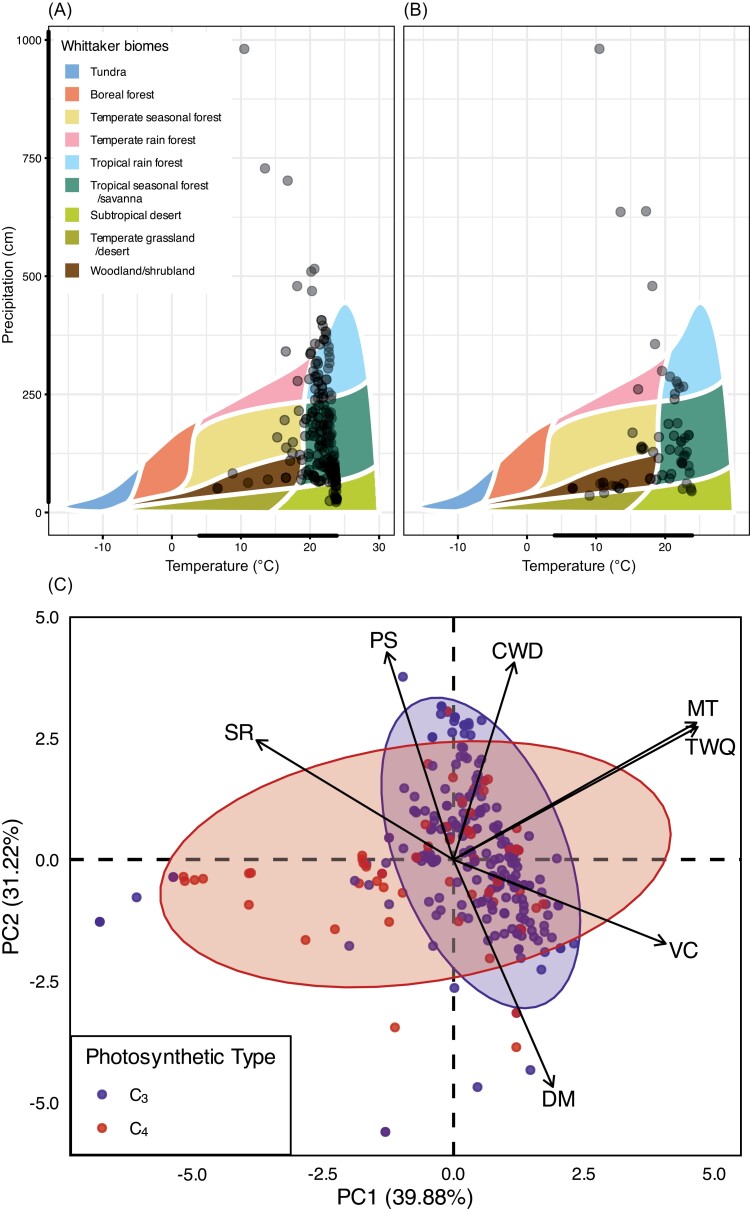
Ecological distributions of C_3_ and C_4_ trees across the Hawaiian Islands. (A) C_3_ and (B) C_4_ trees plotted on the Whittaker Biomes. The thick black bar on each axis shows the range of values for mean annual temperature and precipitation found on the Hawaiian Islands according to the Climate of Hawaii and Rainfall Atlas of Hawaii (Giambelluca *et al*., 2013, 2014). (C) Principal component analysis of seven environmental variables for C_3_ (blue) and C_4_ (red) trees. Arrows show the loading of each of the seven variables. Abbreviations are as follows: MT, minimum temperature of the coldest month; TWQ, temperature of the wettest quarter; DM, precipitation of the driest month; PS, precipitation seasonality; SR, solar radiation; VC, vegetation cover; CWD, climatic water deficit. The 95% confidence ellipses were calculated for each group.

Overall, trees in Euphorbiaceae are broadly distributed across precipitation regimes that span the range found across the Hawaiian Islands (Fig. 2A, B). C_3_ trees occur in areas with as little as 232 mm mean annual precipitation (MAP) on the northwest coast of Hawai`i and as high as 9010 mm MAP in Kaua`i. Similarly, C_4_ trees have been recorded on the south slopes of Mauna Kea in Hawai`i with only 356 mm MAP, but also in Kaua`i with MAP up to 9010 mm. The distribution of Euphorbiaceae trees across temperatures was narrower, but also reflected that of the Hawaiian Islands (Fig. 2A, B). Mean annual temperature (MAT) ranged from 6.6 to 23.8 °C, with the majority of trees occurring within a much narrower range of 18–24 °C. While C_3_ and C_4_ trees inhabited similar temperature spaces, C_3_ trees were more skewed towards the upper end of the temperature range compared with C_4_ trees. According to the Whittaker Biomes theory (Whittaker, 1975), these MAP and MAT ranges suggest that Hawaiian Euphorb trees are most commonly found within tropical rainforest and tropical seasonal forest/savanna biomes. C_3_ and C_4_ trees are similarly distributed across these biomes (Fig. 2A, B).

### C_4_ photosynthesis expands the ecological range of trees in Euphorbiaceae on the Hawaiian Islands

We performed three PCAs to determine which environmental and soil variables most strongly distinguish the distributions of C_3_ and C_4_ individuals on the Hawaiian Islands. The first PCA incorporated the seven environmental explanatory variables that we hypothesized would differ in C_3_ and C_4_ trees based on the published literature on monocots and herbaceous eudicots (Table 2). This generated three PCs which captured nearly 85% (39.88% PC1, 31.22% PC2, and 13.31% PC3) of the variation across the dataset (Fig. 2C; Supplementary Fig. S8). Wettest quarter temperature, minimum coldest month temperature, vegetation cover, and solar radiation were most strongly correlated with PC1, while precipitation seasonality, precipitation of the driest month, and CWD were most strongly correlated with PC2 (Fig. 2C; Supplementary Table S3). The two temperature variables showed strong positive correlation with each other in the PCA space, while the pair of precipitation variables showed strong negative correlation with each other, as did vegetation cover and solar radiation. There is a large degree of overlap between the C_3_ and C_4_ groups in the PCA space, with the 95% confidence ellipse for the C_3_ group almost completely contained within that of the C_4_ group. The C_3_ and C_4_ groups occupied a similar range of values on PC2 but the C_4_ group had a much broader distribution than the C_3_ group on PC1 with a skew towards lower PC1 values. This indicates that C_4_ trees occupy a broader range of ecological niches than C_3_ trees, and that environments with low temperature and vegetation cover, and high levels of solar radiation are more often occupied by C_4_ trees than their C_3_ counterparts.

To determine whether the difference in ecological space between C_3_ and C_4_ trees was larger or smaller than that of closely related herbaceous and shrub life forms, we performed a second PCA on the same seven environmental variables but including all growth forms (herb, shrub, and tree) (Supplementary Figs S5, S6). PC1 accounted for 36.18% of the variation in the dataset, while PC2 accounted for 34.23% (Supplementary Fig. S5). The results of this analysis show that, similarly to trees, C_4_ shrubs occupy a broader range of ecological niches than C_3_ shrubs (Supplementary Fig. S5C). The niche-broadening effect is even more pronounced in shrubs than in trees, and is driven by all of the seven environmental variables. As such, when considered together, C_4_ woody species (i.e. shrubs and trees) generally occupy a greater ecological niche than their C_3_ counterparts (Supplementary Fig. S5E). Conversely, C_3_ and C_4_ non-woody species (i.e. herbs) in this family have a smaller degree of ecological difference than shrubs and trees (shown by their more similar distributions in the PCA space), but C_4_ herbs have a slightly broader range of values for PC2 (and to a lesser extent PC1) compared with C_3_ herbs (Supplementary Fig S5B). PC2 is most strongly correlated with solar radiation, vegetation cover, and precipitation of the driest month (Supplementary Table S4), so this suggests that C_4_ herbs occupy brighter, more open, drier environments than their C_3_ counterparts.

The third PCA incorporated all the continuous soil variables available from the Hawaii Soil Atlas for C_3_ and C_4_ tree species (Supplementary Fig. S7). PC1 accounted for 35.7% of the variation in the dataset, while PC2 accounted for 29.95%. The variables of OM, *K*_sat_, and pH were most strongly correlated with PC1, while CEC was most strongly correlated with PC2 (Supplementary Table S5). There was almost complete overlap of the 95% confidence ellipses for the C_3_ and C_4_ groups in the PCA space (Supplementary Fig. S6), indicating that soil variables did not differ greatly between the C_3_ and C_4_ trees in this dataset.

### C_4_ trees expanded their ecological range more than C_3_ trees as they dispersed across the Islands

**Fig. 3.**
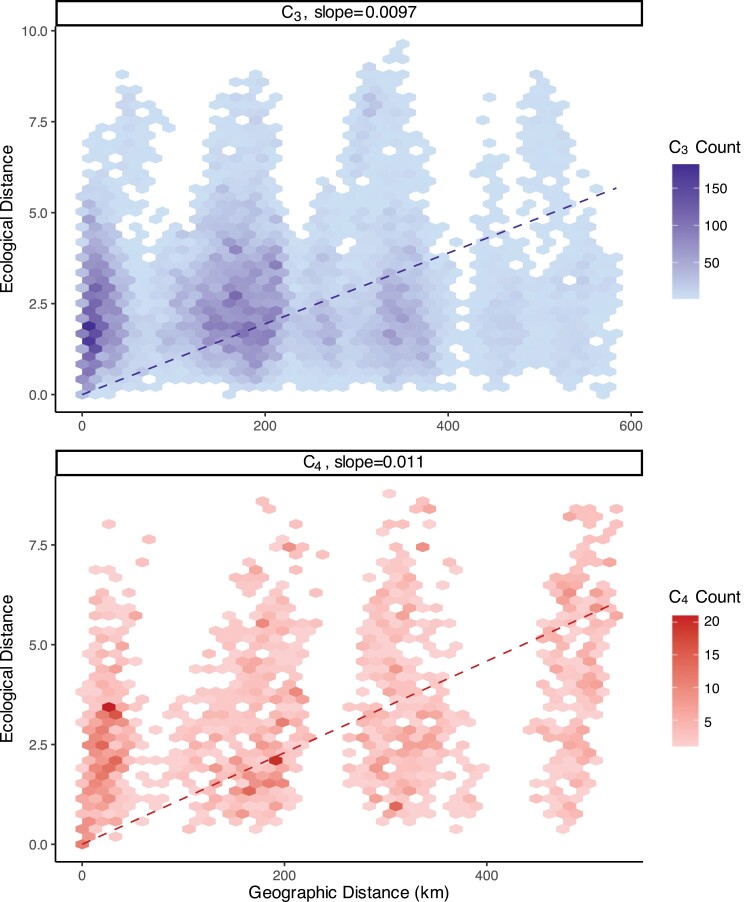
Comparison of geographical and ecological distances for C_3_ and C_4_ trees across the Hawaiian Islands. Ecological and geographical distances were obtained for pairs of C_3_ (blue) and C_4_ (red) individuals. Regression lines forced to the origin were identified for each of the C_3_ and C_4_ groups, and the slopes calculated.

We used Mantel permutation tests to examine the association between ecological and geographical expansion in C_3_ and C_4_ trees. The results of the Mantel tests show a positive correlation between ecological and geographical expansion for both C_3_ (*P*=0.019) and C_4_ (*P*<0.001) trees. This was confirmed by linear regression analysis, which showed that the regression line for the C_4_ group had a slightly steeper slope (0.011) than the C_3_ group (0.0097), indicating that C_4_ trees expanded their ecological niche more for a given degree of geographic dispersal compared with C_3_ trees (Fig. 3). This is possibly associated with a small number of C_4_ trees accessing higher elevation environments, where they are disproportionately abundant relative to C_3_ trees (Fig. 1B).

### Evolutionary history does not strongly influence C_3_ and C_4_ tree distributions

**Fig. 4.**
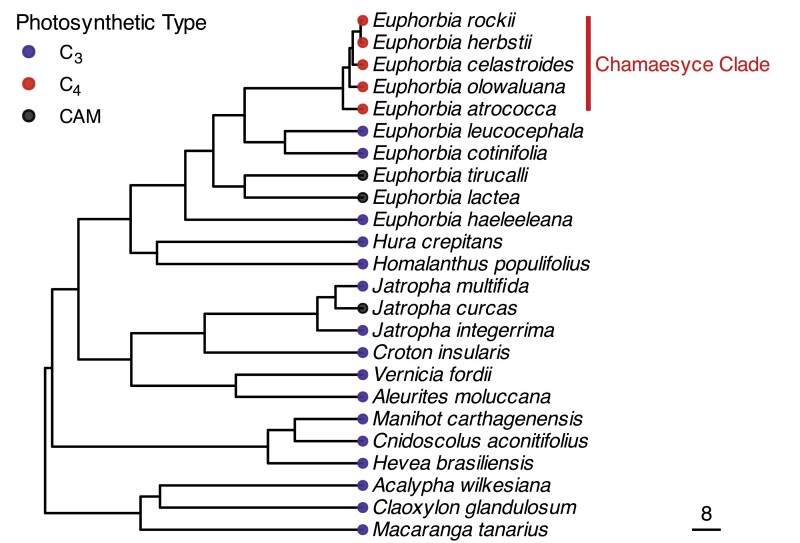
Phylogeny of Hawaiian Euphorbiaceae trees. Evolutionary relationships between C_3_ (blue, *n*=16), C_4_ (red, *n*=5), and CAM (black, *n*=3) Hawaiian Euphorbiaceae trees are presented in a pruned maximum credibility tree inferred from the BEAST analysis of a dataset representing 112 species in Euphorbiaceae. The C_3_ species *Macaranga mappa* is missing from this tree due to an absence of available published nucleotide data. The Hawaiian species *Claoxylon sandwicense* is represented by the congeneric species *C. glandulosum*. The Hawaiian species *Croton guatemalensis* is represented by the congeneric species *C. insularis*.

We generated a phylogeny of Euphorbiaceae trees found on the Hawaiian Islands using previously published sequence data (Fig. 4). Our phylogeny showed that C_4_ tree species on the Hawaiian Islands form a monophyletic group, consistent with previous phylogenies [Fig. 4; Yang *et al.*, 2018 (posterior probability=1, bootstrap=100)]. This is expected given that the Hawaiian Chamaesyce radiation was previously reported as being from a single common ancestor. We used the phylogeny to perform PGLS analyses on each environmental variable and the first three PCs of the first PCA (which included only tree species) to support the findings of the PCA and determine the role of evolutionary history in C_3_ and C_4_ tree environmental distributions. These analyses showed no influence of evolutionary history in six of the seven environmental variables and two of the three PCs, and that none of the environmental variables or PCs showed significant differences between photosynthetic types after accounting for any phylogenetic signal (Table 3). The temperature variables did, however, show marginally significant differences between photosynthetic types. The PGLS showed evidence of strong phylogenetic signal in CWD and PC2 (λ=0.954 and λ=1.000, respectively); however, these variables did not show significant differences between photosynthetic type.

**Table 3.** Results of phylogenetic generalized least squares (PGLS) analysis and ANOVA for the effects of photosynthetic type on environmental variables and principal components (PCs) for C_3_ and C_4_ trees on the Hawaiian Islands.

Parameter ^*a*^	Unit	C_3_ min	C_3_ max	C_3_ mean	C_3_ SD	C_4_ min	C_4_ max	C_4_ mean	C_4_ SD	λ ^*b*^	*F* ^*c*^	P ^*c*^
Minimum Temperature of the Coldest Month	°C	0.410	17.941	15.649	2.983	0.410	17.939	12.477	5.137	0.000	3.971	*0.061*
Temperature of the Wettest Quarter (Growing Season)	°C	4.710	24.565	20.326	3.030	4.710	23.106	17.203	5.103	0.000	3.763	*0.067*
Precipitation of the Driest Month	mm	0.808	611.644	93.195	90.269	4.556	611.644	82.623	107.192	0.000	0.002	0.968
Precipitation Seasonality	–	0.101	1.058	0.356	0.196	0.128	0.774	0.371	0.161	0.000	0.058	0.812
Yearly Average Solar Radiation	W m^–2^	148.715	290.951	202.409	26.206	152.292	290.951	216.390	30.964	0.057	0.285	0.600
Yearly Average Vegetation Cover Fraction	–	0.048	1.000	0.736	0.205	0.048	0.991	0.674	0.272	0.000	0.043	0.839
Climatic Water Deficit	mm	504.600	6620.157	2665,233	1502.165	504.600	4784.676	2072.157	951.226	0.954	0.008	0.930
PC1	–	–6.774	2.311	0.290	1.285	–6.774	1.720	–0.993	2.301	0.000	2.421	0.136
PC2	–	–5.603	3.764	0.132	1.526	–5.603	3.058	–0.287	1.366	1.000	0.484	0.495
PC3	–	–2.422	2.216	0.053	1.009	–2.091	1.383	–0.111	0.882	0.000	0.248	0.624

^*a*^ Minimum (min), maximum (max), mean, and SD are given for each of seven environmental variables and three PCs for C_3_ (*n*=184 individuals, 16 species) and C_4_ (*n*=66 individuals, five species) trees.

^*b*^ Pagel’s lambda (λ) was obtained from the PGLS and estimates the variance due to phylogenetic sources.

^*c*^ *F*- and *P-*values were obtained from the ANOVA. Marginally significant results (*P*<0.1) are italicized. No variable remained significant, marginal or otherwise, after a Bonferroni correction.

All values are given to three decimal places.

## Discussion

### C_3_ and C_4_ trees have more similar geographical and environmental distributions than C_3_ and C_4_ herbaceous species

The results presented here suggest that C_3_ and C_4_ tree species across the Hawaiian Islands inhabit similar geographical and environmental spaces. There has been no significant niche shift after the single origin of the C_4_ pathway in this clade, as indicated by the paucity of phylogenetic signal in the data. Where phylogenetic signal was present, in CWD and PC2, this was not associated with a significant difference between the two photosynthetic types. This suggests that this phylogenetic structuring is present across the whole family and is not driven by a niche shift associated with the evolution of C_4_ photosynthesis.

The similar ecological distributions of C_3_ and C_4_ tree species contrasts with previous studies on herbaceous species from other families and geographical regions, which show clear differences between the ecological niches of C_3_ and C_4_ grasses and forbs (Teeri and Stowe, 1976; Stowe and Teeri, 1978; Pyankov *et al.*, 2010; Bremond *et al.*, 2012; Pau *et al.*, 2013). Given that all true angiosperm trees are eudicots, and, in C_3_ versus C_4_ eudicots, aridity is an important determinant of distribution (more so than temperature, which is the primary determinant for monocots), we hypothesized that aridity would be the primary environmental determinant distinguishing the distributions of C_3_ and C_4_ trees. However, we found no difference in the driest month precipitation, precipitation seasonality, or CWD between C_3_ and C_4_ trees, despite highly variable precipitation levels across the Hawaiian Islands (Fig. 2; Table 3). In fact, precipitation variables seemed to be the least important factors in determining C_4_ tree distribution, with solar radiation, vegetation cover, and temperature driving differences in distributions between the C_3_ and C_4_ groups in the PCA space (Fig. 2). Soil variables were notably similar between the C_3_ and C_4_ groups; however, this is consistent with a previous study on the biogeographic controls on C_3_ and C_4_ grass distributions (Griffith *et al.*, 2015).

There are many possible factors contributing to the similar environmental distributions of C_3_ and C_4_ trees. First, the height of trees relative to herbaceous species may play a role, as tall plant height may confer greater embolism vulnerability under drought (Olson *et al.*, 2018), and this inherent limitation may occur regardless of photosynthetic type. However, the C_4_ trees on the Hawaiian Islands have not been observed to exceed 10 m in height (Pearcy and Troughton, 1975; Sporck, 2011) so this effect is likely to be minor. Second, and more likely, the small geographical area of the Hawaiian Islands (~28 000 km^2^) may also constrain the degree of environmental differences between C_3_ and C_4_ trees. Strong trends in global distribution patterns are observed for C_3_ and C_4_ grasses, where the geographical area in question is much larger: global grassland areas comprise ~52.5 million km^2^ (Sage *et al.*, 1999; White *et al.*, 2000). However, previous studies of C_3_ and C_4_ grasses on the Hawaiian Islands have revealed significant differences in environmental distributions (Edwards and Still, 2008; Still *et al.*, 2014), so while differences in ecological niche may be less pronounced in an island environment, they are not necessarily completely obscured. Third, the nature of the climate and soil datasets may mask some of the true variability in environmental niche. In the case of the Hawaiian climate datasets, the data are averaged over multiple years, and the Hawaiian soil atlas data are only available for a limited set of variables and a limited number (61% in this study) of occurrence data points. Notably, the Hawaiian soil atlas lacks data on soil nitrogen content, although soil nitrogen availability is related to some of the other variables included in this analysis such as CEC (which is linked to NH_4_^+^ levels) and pH and organic matter content (which are linked to nitrate levels) (Kemmitt *et al.*, 2006). Soil nitrogen availability to plants may also be influenced by temperature, slope, soil aeration, and soil water (Amer and Bartholomew, 1951; Stanford *et al.*, 1975; Kong *et al.*, 2019), all of which vary across the Islands. It is possible that soil nitrogen availability may affect the distribution of the different photosynthetic types in this study given that the C_4_ pathway increases nitrogen use efficiency (Pearcy and Ehleringer, 1984), but this effect cannot be detected in our analyses. However, this is likely to be a limitation for most studies of this nature, many of which still show clear differences in the ecological niches of C_3_ and C_4_ herbaceous species. Finally, life history is probably also significant. Grasses can have both annual and perennial life history strategies, and it is annual grasses (particularly C_4_ annuals) which grow in the hottest environments with the most seasonal precipitation, whereas perennial grasses tend to grow in regions with lower, more seasonal precipitation (Liu *et al.*, 2019). This indicates that C_4_ photosynthesis may be more important for carbon accumulation in hot, dry environments for fast-lived grasses, but not as important in longer lived species such as trees.

### C_4_ trees occupy a broad ecological niche on highly heterogenous Islands

Although C_3_ and C_4_ trees occupy similar environments, the results of the PCA and linear regression analysis of the environmental versus geographical distances suggest that C_4_ photosynthesis does have a niche-broadening effect across all growth forms in Euphorbiaceae, with this effect being the most pronounced in woody species (Figs 2, 3; Supplementary Fig. S5). In particular, C_4_ tree species have expanded their range into environments with characteristically sparse vegetation cover, higher sunlight, and cooler temperatures (Fig. 2C). Reduced vegetation cover, increased solar radiation, and cooler temperatures are all correlated with increasing elevation levels (Supplementary Fig. S9). This suggests that C_4_ trees have expanded their ecological niche relative to their C_3_ counterparts as they moved into higher elevation environments. It is worth noting that this niche broadening is not an effect of different sample sizes as there are fewer C_4_ than C_3_ tree occurrence points, and broader ecological distributions of C_4_ species are seen in all life forms in this study, despite differences in relative sample sizes of C_3_ versus C_4_ groups. Furthermore, a broadening of the environmental niche associated with C_4_ photosynthesis is consistent with a previous intraspecific study in grasses (Lundgren *et al.*, 2015). As such, it may be that C_4_ photosynthesis does influence the niche of woody species on the Hawaiian Islands, but that there has not been sufficient opportunity for niche specialization among C_4_ tree species to generate an apparent niche shift relative to their C_3_ counterparts due to their restricted global range. As previously stated, the small geographic range of C_4_ trees could have acted to limit the environmental conditions that they can access, even though this limitation is minimized by two factors.

First, the trees have dispersed across six of the Hawaiian Islands (Fig. 1), which gives them the opportunity to access the full range of the environments and climates that occur across the Islands (Fig. 2). This dispersal across the Islands has probably been facilitated by the seed characteristics of the ancestor of the C_4_ Hawaiian *Euphorbia*, which had small seeds with mucilaginous seed coats that adhered to birds to facilitate dispersal (Price and Wagner, 2004). Some species of Hawaiian *Euphorbia* have then undergone habitat specialization following dispersal, such as the C_4_ tree *E. rockii*, which is a single-island endemic on O`ahu (Yang *et al.*, 2018). *Euphorbia rockii* has large, non-sticky seeds which may be beneficial for seedling survival in forest understorey habitats, but also have reduced dispersal (Koutnik, 1987; Jordan and Hayden, 1992; Yang *et al.*, 2018). It is worth noting that there are some islands, such as Ni`ihau, that have no record of Euphorb species (Supplementary Fig. S3), which may be a result of limited sampling rather than true absence.

Second, the environments of the Hawaiian Islands are highly heterogenous, which generates large environmental gradients over relatively small geographic distances (Barajas-Barbosa *et al.*, 2020). This underlies the suitability of the Hawaiian Islands for this type of analysis: there is the potential for large ecological niche variation between species in close geographic proximity and, as such, there is a high degree of potential variation in climatic and environmental variables between individuals. Indeed, studies of the ecological differences between C_3_ and C_4_ grasses on the Islands have identified trends in ecological distributions that are consistent with globally observed patterns (Pau *et al.*, 2013; Still *et al.*, 2014).

Despite these mitigating factors, it is likely that geographical limitation still acts to constrain the magnitude of potential environmental differences between C_3_ and C_4_ trees. As the Hawaiian Islands formed, this generated a high availability and diversity of new niches, which probably facilitated the radiation of the C_4_*Euphorbia* (Yang *et al.*, 2018). However, the small and isolated nature of the Hawaiian Islands means that the potential for niche shifts following this initial radiation is likely to be lower than that on a continental land mass. This is reflected in the limited environmental differences that are seen across all growth forms (Supplementary Fig. S5) in Euphorbiaceae on the Hawaiian Islands. Indeed, herbaceous C_3_ and C_4_ Hawaiian Euphorbs seem to grow in more similar environments than has previously been observed for their counterparts in other geographical regions (Batanouny *et al.*, 1991).

### Euphorbiaceae is a morphologically and photosynthetically diverse plant family

In addition to the geographic limitations of the Hawaiian Islands, there are further complications in this study in conclusively determining growth form and photosynthetic type in Euphorbiaceae.

With respect to growth form, there is no unilaterally accepted definition of a tree: it is not a single phylogenetic grouping, but a life ‘strategy’ that can vary between and within species. For example, *Euphorbia celastroides* can achieve the tree growth form, but also has varieties with shrubbier, more prostrate growth forms (Sporck, 2011; Yang *et al.*, 2018), and distinguishing between a large shrub and a small tree of the same species is subjective. These intraspecific differences are not resolved within the available occurrence data, so certain individuals identified as trees in this study may have had shrubbier growth forms. However, this issue is mitigated somewhat by the fact that the ecological differences between C_3_ and C_4_ shrubs and trees seem to be driven by similar factors, and the niche-broadening effect of C_4_ photosynthesis is consistent across all woody species (Supplementary Fig. S5).

Furthermore, photosynthetic type has not been conclusively established for all the species in Euphorbiaceae, as determining photosynthetic type can require a combination of carbon isotope discrimination (δ^13^C), gas exchange, and leaf anatomy data. These data are not available in the literature for all species: for most species, only δ^13^C data could be obtained. As such, there may be unrecognized photosynthetic diversity within species currently classified as using C_3_ photosynthesis. This is made more likely by the fact that Euphorbiaceae is a highly photosynthetically diverse plant family, known to include species using C_3_, C_4_, C_3_–C_4_ intermediate, and CAM modes of photosynthesis, although there are currently no known C_3_–C_4_ trees in Euphorbiaceae or otherwise.

### Photosynthetic diversity in Euphorbiaceae provides insights into C_4_ evolution in tree species

The reason for the global rarity of C_4_ and C_3_–C_4_ tree species is an ongoing question in plant ecophysiology and evolution. In order to answer this question, the two potential evolutionary routes to C_4_ photosynthesis in trees must be assessed (Young *et al.*, 2020).

First, C_4_ photosynthesis may arise in an existing tree, via a C_3_–C_4_ intermediate state. The feasibility of this evolutionary path cannot be confirmed due to the apparent absence of C_3_–C_4_ trees from global plant biodiversity. Given the high degree of photosynthetic diversity in woody Euphorbs, the lack of C_3_–C_4_ photosynthesis is particularly apparent. Indeed, in *Euphorbia* alone, there have been at least 17 independent evolutions of a photosynthetic CO_2_-concentrating mechanism; 16 evolutions of CAM and one of C_4_ (Horn *et al.*, 2014). The C_4_ lineage was ancestrally herbaceous, while all 16 evolutions of CAM occurred in ancestrally woody lineages. In view of this, Horn *et al.* (2014) hypothesize that life history is important in establishing evolutionary trajectory. We hypothesize that biogeographical factors associated with life history traits may also affect the ability of a lineage to evolve C_4_ photosynthesis; that is, a lower quantum yield in C_3_–C_4_ photosynthetically intermediate species (i.e. potential C_3_–C_4_ trees) could occlude them from shaded habitats (Monson *et al.*, 1986; Monson and Moore, 1989). This could therefore select against the evolution of intermediate photosynthetic types, and thus C_4_ photosynthesis, in ancestrally woody forest species, and provides some explanation for the absence of woody C_3_–C_4_ species.

Second, a C_4_ tree species may evolve from an herbaceous C_4_ ancestor. This was the route taken by the Hawaiian *Euphorbia*, indicating that this is a feasible evolutionary path (Yang *et al.*, 2018). However, if the transition to the tree state means that the potential benefits of the C_4_ pathway are diminished or eliminated, this reduces the likelihood of C_4_ trees emerging via this pathway and persisting in competitive environments, thus providing some explanation for the rarity of C_4_ trees. The minimal ecological differences between C_3_ and C_4_ trees presented here offer some support for this explanation: there may be little to no benefit, or even a cost, to the C_4_ photosynthetic pathway in trees in terms of carbon or water gain in hotter and/or drier climates. It is possible that C_4_ photosynthesis has only persisted in trees on the Hawaiian Islands due to the high availability and diversity of new niches that emerged as the Islands formed (which has been shown to be consistent with a high level of species diversity; Sebastian *et al.*, 2012; Barajas-Barbosa *et al.*, 2020), rather than the C_4_ photosynthetic pathway providing any benefit over the ancestral C_3_ state. However, it is difficult to fully assess the validity of this conclusion given that the Hawaiian *Euphorbia* represent a single C_4_ lineage (one of 34 in the eudicots), and the only C_4_ lineage to include true trees (Sage, 2016).

If indeed it is the case that the C_4_ pathway provides limited benefit in trees, then this not only helps to explain why C_4_ photosynthesis is so rare in trees but also provides insight into the value (or lack thereof) of C_4_ trees from an engineering perspective. If the C_4_ pathway did allow trees to perform better under hot and/or dry environmental conditions, the engineering of C_4_ photosynthesis into trees would be a valid target for future-proofing against climate change to secure timber yields or forest carbon sequestration. However, if the C_4_ pathway does not influence tree ecology in this way, as the results presented here suggest, then this approach would not be beneficial. C_4_ trees may only be exceptional with respect to their rarity, and not their performance relative to their C_3_ counterparts.

### Conclusions

Our results suggest that C_3_ and C_4_ trees in Euphorbiaceae inhabit similar environments across the Hawaiian Islands. This may, in part, be due to the limited geographic range of C_4_ trees globally, but may also indicate that C_4_ photosynthesis is not beneficial in trees in terms of accessing hotter and/or drier climates. However, C_4_ photosynthesis does appear to have a niche-broadening effect in tree species, and C_4_ trees have expanded their range on the Hawaiian Islands into environments with characteristically sparse vegetation cover, higher sunlight, and cooler temperatures. The high level of photosynthetic and morphological diversity in Euphorbiaceae merits further investigation to conclusively establish (i) how C_4_ photosynthesis, in this rare case, evolved in trees; (ii) whether there has been evolution of intermediate photosynthetic types in woody species in this family; and (iii) whether these intermediate photosynthetic types may confer any benefit over the C_3_ state.

## Supplementary data

The following supplementary data are available at *JXB* online.

Fig. S1. Map of occurrence data flagged by CoordinateCleaner.

Fig. S2. Map of occurrence data by data source.

Fig. S3. Map of distributions of Hawaiian herbs, shrubs, and trees by photosynthetic type.

Fig. S4. Complete phylogeny of all species whose data were obtained in this study.

Fig. S5. Principal components 1 and 2 of a principal component analysis (PCA) of environmental variables for all growth forms.

Fig. S6. Principal components 3 and 4 of a principal component analysis (PCA) of environmental variables for all growth forms.

Fig. S7. Principal component analysis (PCA) of soil variables for C_3_ and C_4_ trees.

Fig. S8. Histogram of principal component 3 of a principal component analysis (PCA) of environmental variables for C_3_ and C_4_ trees.

Fig. S9. Distribution of C_3_ and C_4_ trees by elevation and environmental variables correlated with elevation.

Table S1. Issues identified by GBIF which resulted in a record being removed from this analysis

Table S2. Summary of sequence data used and accession numbers.

Table S3. Environmental variables correlated to PCA axes (trees only).

Table S4. Environmental variables correlated to PCA axes (all life forms).

Table S5. Soil variables correlated to PCA axes (trees only).

erac113_suppl_Supplementary-Material

## Data Availability

The data that were used in this study were all from publicly available datasets which have been fully cited.
