# Supplementary material for: C4 trees have a broader niche than their close C3 relatives
Source: J Exp Bot. 2022 May 23;73(10):3189–204. doi: 10.1093/jxb/erac113 (PMC9126736; doi:10.1093/jxb/erac113)
Supplement: erac113_suppl_Supplementary-Material [file erac113_suppl_supplementary-material.pdf]

Supplementary material for:

C_4 trees have broader niches than their close C_3 relatives

Sophie N. R. Young, Luke T. Dunning, Hui Liu, Carly J. Stevens, Marjorie R. Lundgren

CONTENTS:

Supplementary Figures

Fig S1. Map of occurrence data flagged by CoordinateCleaner

Fig S2. Map of occurrence data by data source

Fig S3. Map of distributions of Hawaiian herbs, shrubs and trees by photosynthetic type

Fig S4. Complete phylogeny of all species whose data was obtained in this study

Fig S5. Principal components 1 and 2 of a Principal Component Analysis (PCA) of environmental variables for all growth forms

Fig S6. Principal components 3 and 4 of a Principal Component Analysis (PCA) of environmental variables for all growth forms

Fig S7. Principal Component Analysis (PCA) of soil variables for C_3 and C_4 trees

Fig S8. Histogram of principal component 3 of a Principal Component Analysis (PCA) of environmental variables for C_3 and C_4 trees.

Fig S9. Distribution of C_3 and C_4 trees by elevation and environmental variables correlated with elevation.

Supplementary Tables

Table S1. Issues identified by GBIF which resulted in a record being removed from this analysis

Table S2. Summary of sequence data used and accession numbers

Table S3. Environmental variables correlated to PCA axes (trees only)

Table S4. Environmental variables correlated to PCA axes (all lifeforms)

Table S5. Soil variables correlated to PCA axes (trees only)

SUPPLEMENTARY FIGURES

Figure S1

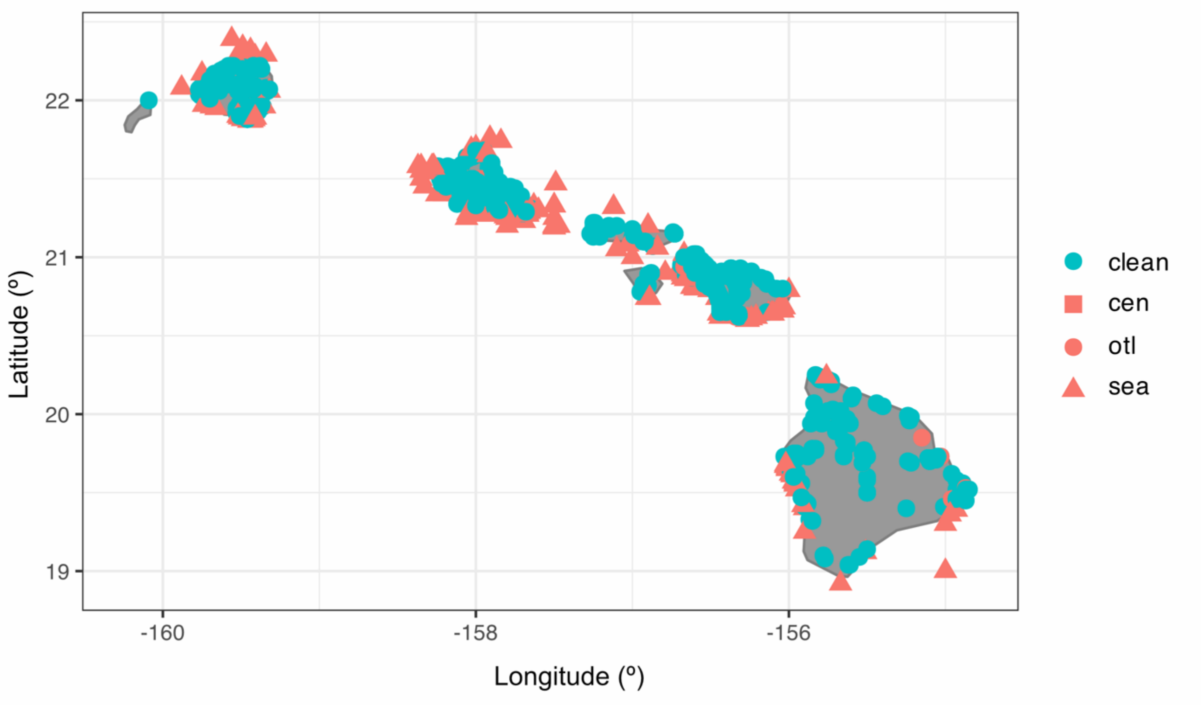

***Fig S1*. Map of occurrence data flagged by CoordinateCleaner.** Occurrence data for species in Euphorbiaceae on the Hawaiian Islands were processed using the CoordinateCleaner package in R. 277 coordinates were flagged (pink) including those located on country centroids (cen, n = 1, squares), species outliers (otl, n = 22, circles), or in the sea (sea, n = 262, triangles), and removed prior to further analysis. Remaining coordinates (n = 1091) are designated as 'clean' (blue circles).

Figure S2

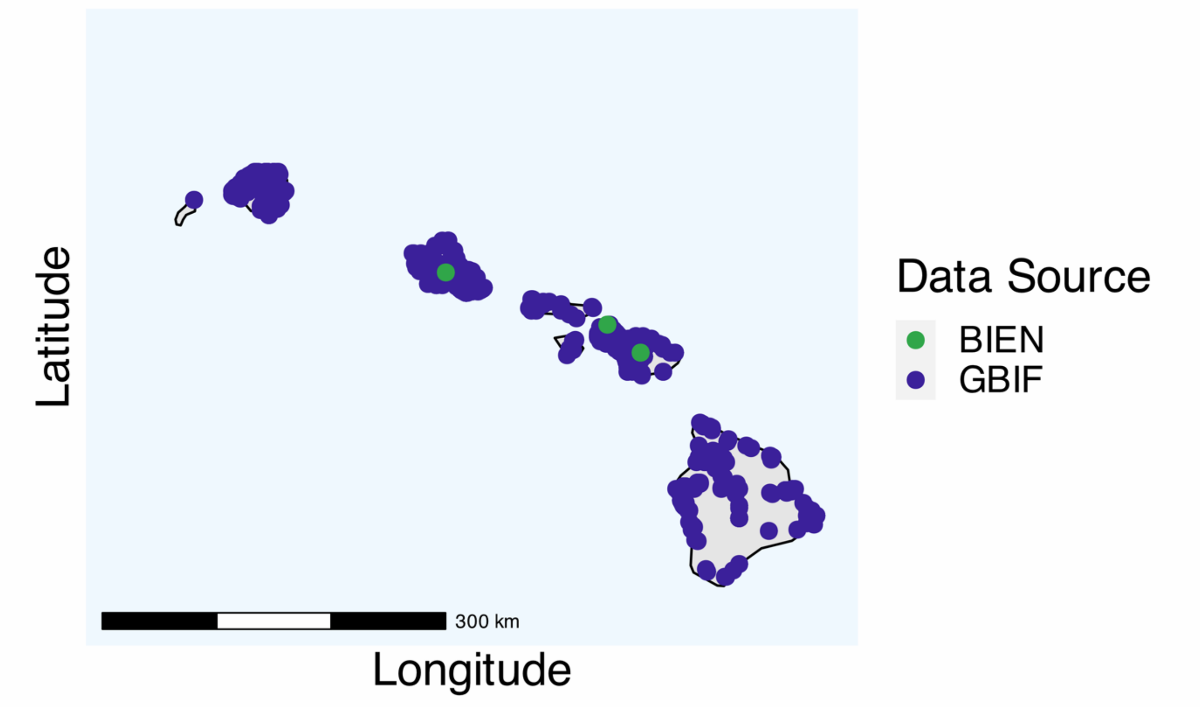

Fig S2. **Map of occurrence data by data source.** Cleaned occurrence data are shown for species in Euphorbiaceae that were obtained from either the Botanical Information and Ecology Network (BIEN, n = 3, green) or the Global Biodiversity Information Facility (GBIF, n = 687, blue). Duplicate records (*i.e.* those that appeared in both datasets, or twice in one dataset) have been removed.

Figure S3

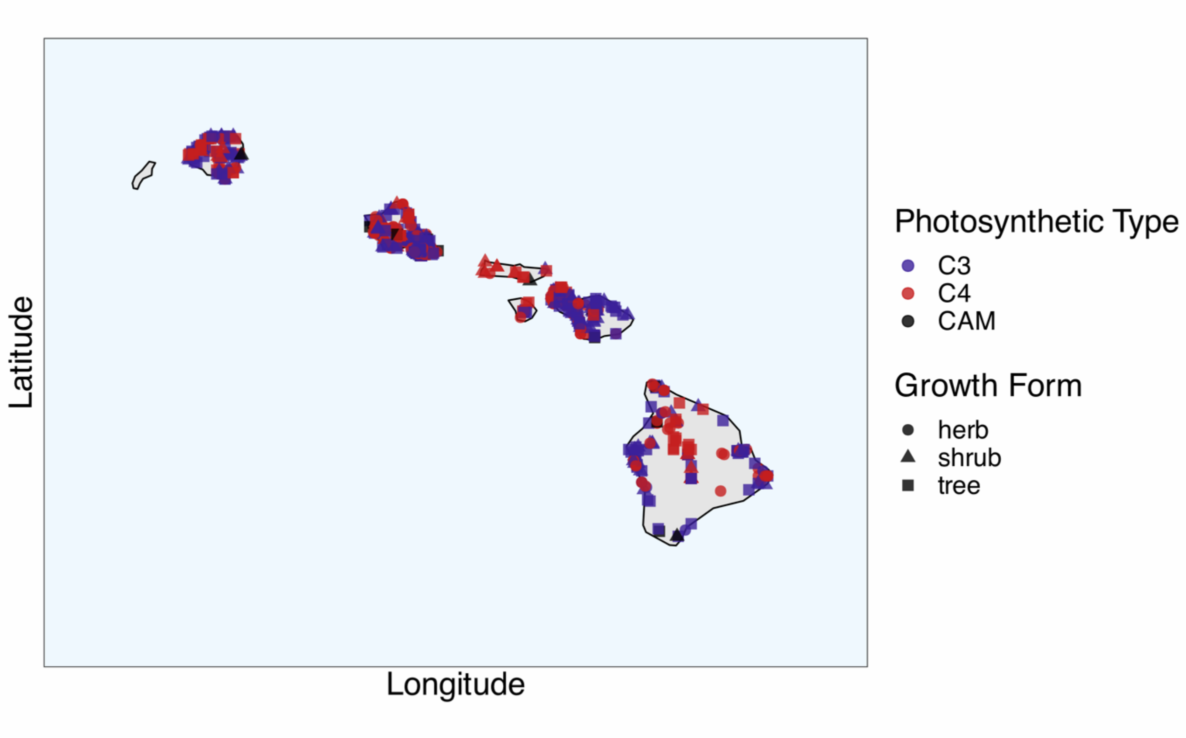

***Fig S3*. Map of distributions of Hawaiian herbs, shrubs and trees by photosynthetic type.** Occurrence data for herbs (n = 199, representing 9 species, circles), shrubs (n = 170, representing 18 species, triangles) and trees (n = 283, representing 25 species, squares) in Euphorbiaceae are shown on a map of the Hawaiian Islands. Points are coloured according to the photosynthetic type assigned to each species: C_3 (blue), C_4 (red) or CAM (black).

Figure S4

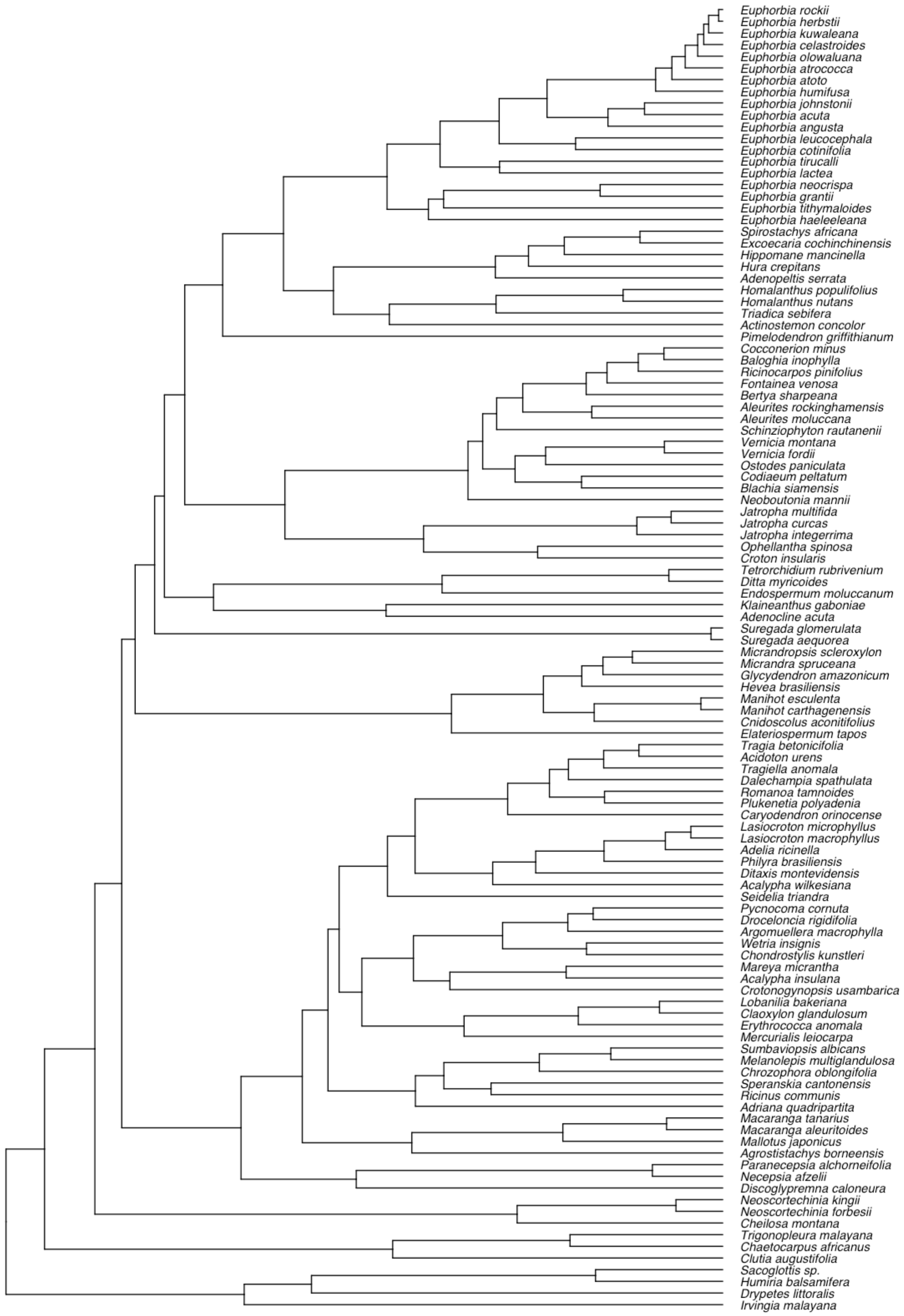

***Fig S4*. Complete phylogeny of all species whose data was obtained in this study.** Phylogenetic tree generated using previously published data for 112 species in Euphorbiaceae.

Figure S5

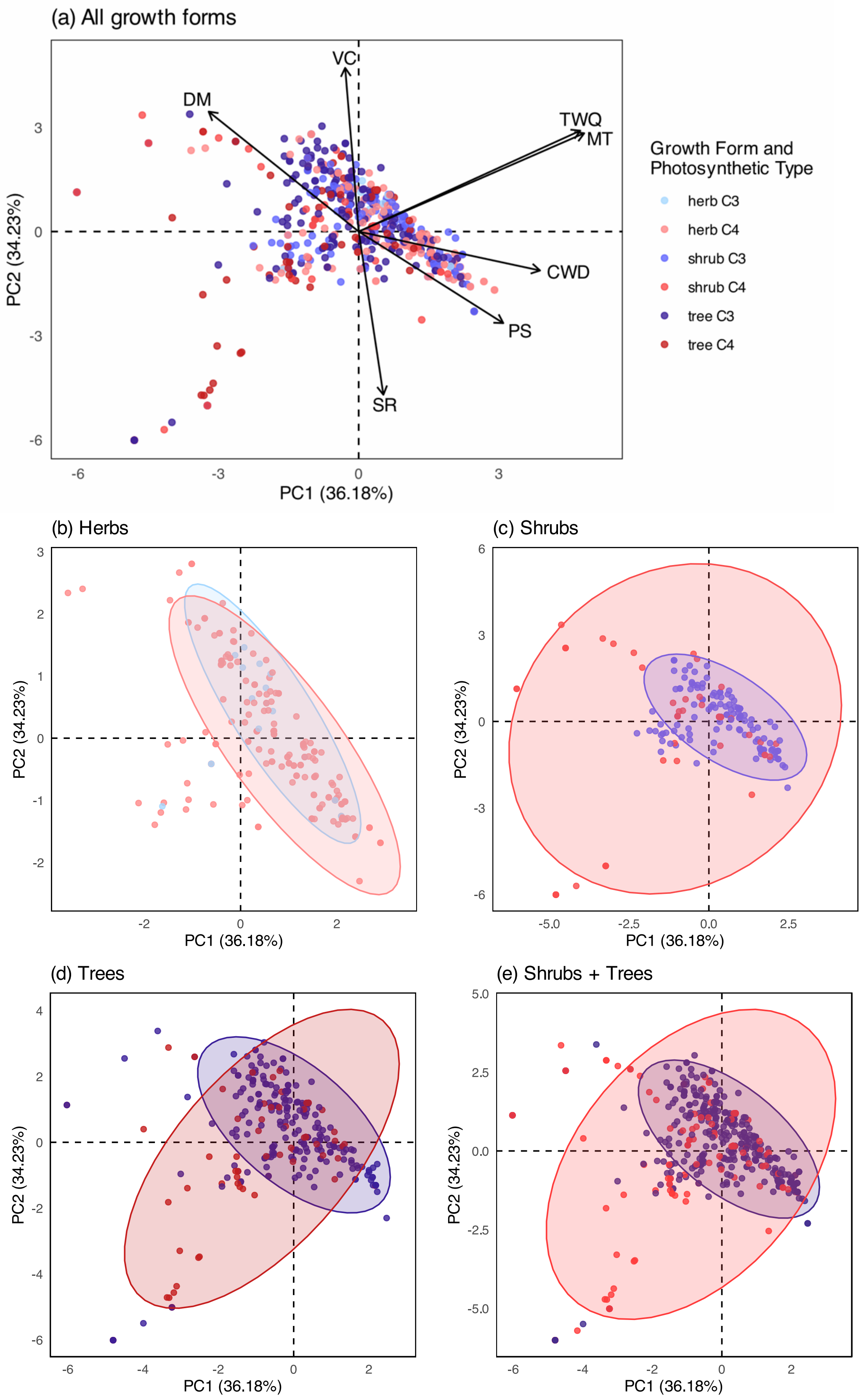

(a) All growth forms
VC
DM
TWQ
MT
CWD
PS
SR
PC2 (34.23%)
PC1 (36.18%)
Growth Form and
Photosynthetic Type
herb C3
herb C4
shrub C3
shrub C4
tree C3
tree C4
(b) Herbs
(c) Shrubs
(d) Trees
(e) Shrubs + Trees

***Fig S5.* Principal components 1 and 2 of a Principal Component Analysis (PCA) of environmental variables for all growth forms.** (a) PCA plot showing C_3 (blue) and C_4 (red) herb, shrub, and tree species in Euphorbiaceae. Arrows show the loading of each of the 7 environmental variables used in the PCA. Abbreviations are as follows: MT=minimum temperature of the coldest month, TWQ= temperature of the wettest quarter, DM=precipitation of the driest month, PS=precipitation seasonality, SR=solar radiation, VC=vegetation cover, CWD=climatic water deficit. Data are also shown on individual plots separated by growth form, with 95% confidence ellipses calculated for each group. (b) C_3 (n = 21) and C_4 (n = 178) herbaceous species, (c) C_3 (n = 119) and C_4 (n = 47) shrub species, C_3 (n = 202) and C_4 (n = 66) tree species, and (e) C_3 (n = 331) and C_4 (n = 113) woody (herb and shrub) species.

Figure S6

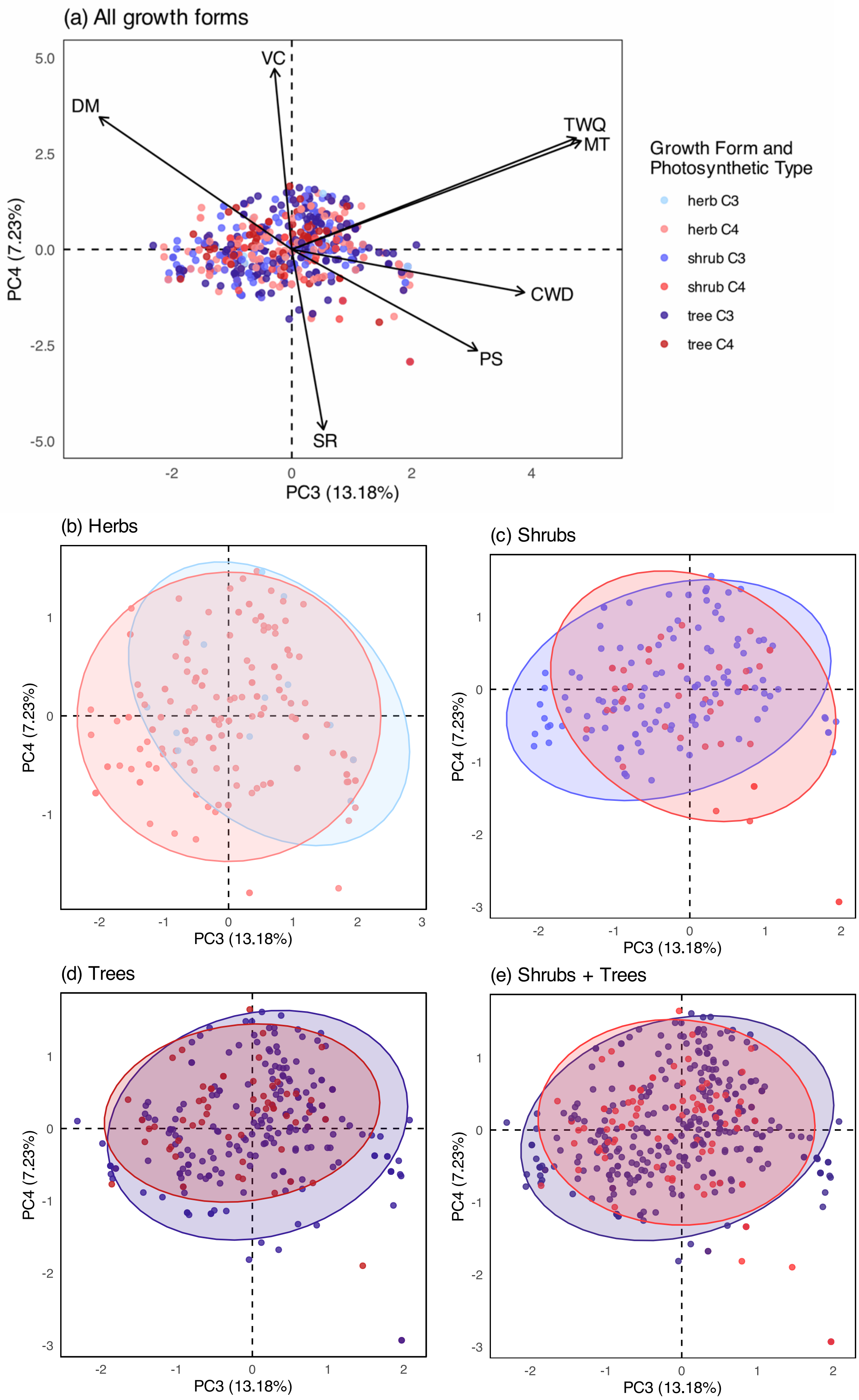
(a) All growth forms
VC
DM
TWQ
MT
CWD
PS
SR
PC4 (7.23%)
PC3 (13.18%)
Growth Form and Photosynthetic Type
herb C3
herb C4
shrub C3
shrub C4
tree C3
tree C4
(b) Herbs
(c) Shrubs
(d) Trees
(e) Shrubs + Trees

Fig S6. **Principal components 3 and 4 of a Principal Component Analysis (PCA) of environmental variables for all growth forms.** (a) PCA plot showing C_3 (blue) and C_4 (red) herb, shrub, and tree species in Euphorbiaceae. Arrows show the loading of each of the 7 environmental variables used in the PCA. Abbreviations are as follows: MT=minimum temperature of the coldest month, TWQ= temperature of the wettest quarter, DM=precipitation of the driest month, PS=precipitation seasonality, SR=solar radiation, VC=vegetation cover, CWD=climatic water deficit. Data are also shown on individual plots separated by growth form, with 95% confidence ellipses calculated for each group. (b) C_3 (n = 21) and C_4 (n = 178) herbaceous species, (c) C_3 (n = 119) and C_4 (n = 47) shrub species, C_3 (n = 202) and C_4 (n = 66) tree species, and (e) C_3 (n = 331) and C_4 (n = 113) woody (herb and shrub) species.

Figure S7

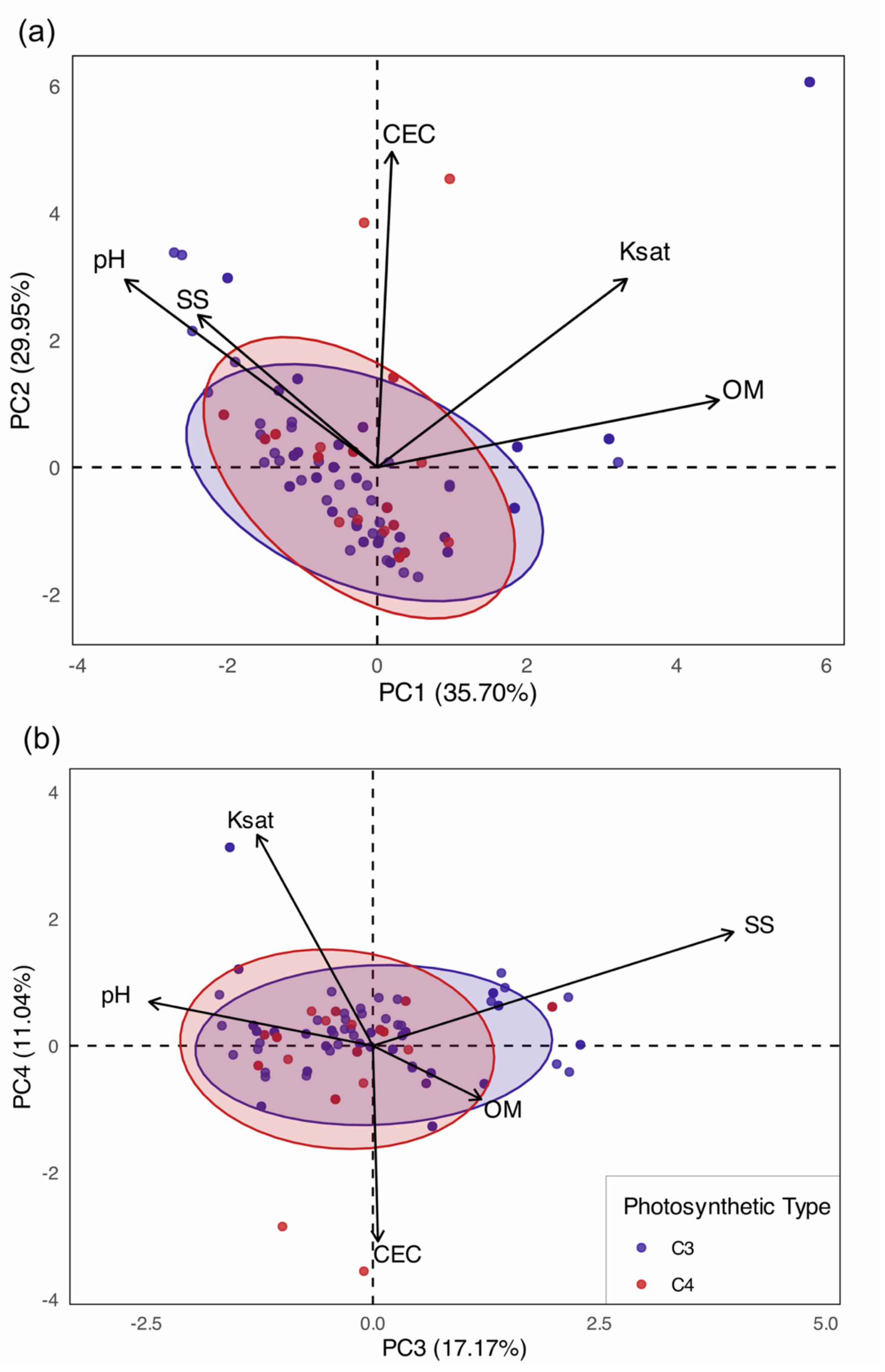

***Fig S7*. Principal component analysis (PCA) of soil variables for C_3 and C_4 trees.** (a) Principal Components (PCs) 1 and 2, and, (b) PCs 3 and 4 of a PCA conducted on 5 soil variables for C_3 (blue) and C_4 (red) trees. Arrows show the loading of each of the 5 variables. Abbreviations are as follows: CEC = cation exchange capacity, Ksat = water permeability, SS = shrink-swell potential (soil stability), OM = organic matter. 95% confidence ellipses were calculated for each group.

Figure S8

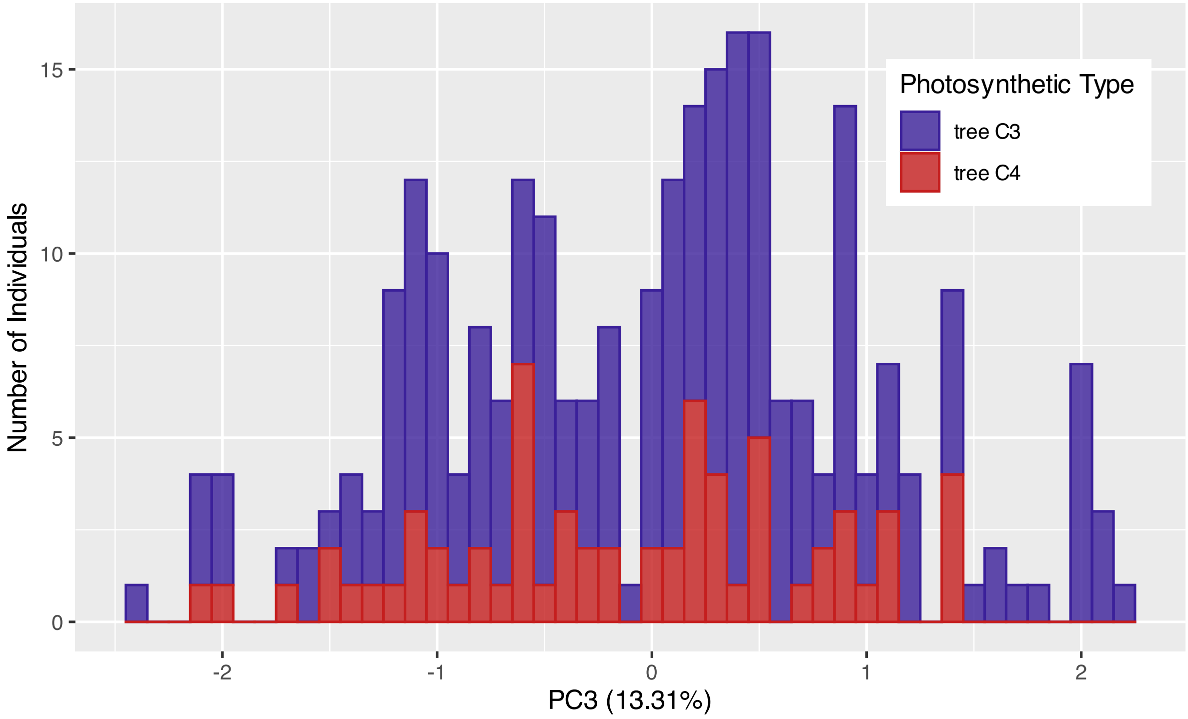

***Fig S8*. Histogram of Principal Component 3 of a Principal Component Analysis (PCA) of environmental variables for C_3 and C_4 trees.** Histogram showing C_3 (blue, n = 202) and C_4 (red, n = 66) tree species in Euphorbiaceae.

Figure S9

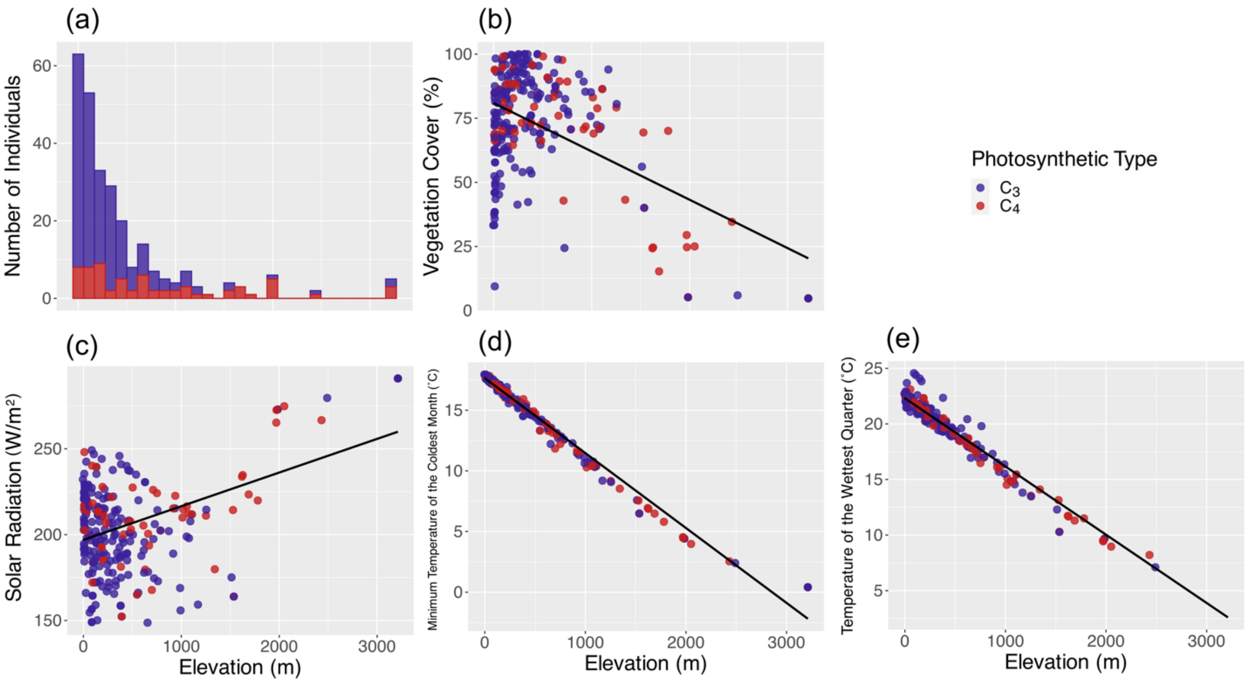

***Fig S9.* Distribution of C_3 and C_4 trees by elevation, and environmental variables correlated with elevation.** (a) Histogram of the distribution of C_3 (blue) and C_4 (red) trees by elevation. Scatterplots of the relationship between elevation and 4 environmental variables are shown: (b) vegetation cover, (c) solar radiation, (d) minimum temperature of the coldest month and (e) temperature of the wettest quarter. Linear regression lines (black) were fitted to the data in plots b-e.

SUPPLEMENTARY TABLES

Table S1. **Issues identified by GBIF which resulted in a record being removed from this analysis**

**code**	**issue**	**description**	**type**
conti	CONTINENT_INVALID	Uninterpretable continent values found.	occurrence
cucdmis	COUNTRY_COORDINATE_MISMATCH	The interpreted occurrence coordinates fall outside of the indicated country.	occurrence
cuiv	COUNTRY_INVALID	Uninterpretable country values found.	occurrence
cum	COUNTRY_MISMATCH	Interpreted country for dwc:country and dwc:countryCode contradict each other.	occurrence
preneglat	PRESUMED_NEGATED_ LATITUDE	Latitude appears to be negated, e.g. 32.3 instead of -32.3	occurrence
preneglon	PRESUMED_NEGATED_ LONGITUDE	Longitude appears to be negated, e.g. 32.3 instead of -32.3	occurrence
preswcd	PRESUMED_SWAPPED_ COORDINATE	Latitude and longitude appear to be swapped.	occurrence
txmatfuz	TAXON_MATCH_FUZZY	Matching to the taxonomic backbone can only be done using a fuzzy, non-exact match.	occurrence
cdpi	COORDINATE_PRECISION_INVALID	Indicates an invalid or very unlikely coordinatePrecision	occurrence

Table S2. summary of sequence data used and accession numbers

	rbcL		matK		trnL		
**Species**	**Length (bp)**	**Accession**	**Length (bp)**	**Accession**	**Length (bp)**	**Accession**	**Total (bp)**
Acalypha_insulana	1331	AB233854	1203	AB233750	0	-	2534
Acalypha_wilkesiana	599	KU243027	0	-	0	-	599
Acidoton_urens	1321	AB267913	1194	AB268017	0	-	2515
Actinostemon_concolor	1324	AB233883	1203	AB233779	0	-	2527
Adelia_ricinella	1315	AB267914	1203	AB268018	1016	AY794737	3534
Adenocline_acuta	1325	AB233874	1197	AB233770	0	-	2522
Adenopeltis_serrata	1324	AB267954	1203	AB268058	791	AY794633	3318
Adriana_quadripartita	1325	AB233855	1206	AB233751	0	-	2531
Agrostistachys_borneensis	1331	AB233856	1203	AB233752	0	-	2534
Aleurites_moluccana	1321	AY794883	1218	LK021377	904	AY794709	3443
Aleurites_rockinghamensis	0	-	0	-	743	KC428437	743
Argomuellera_macrophylla	1325	AB267915	1203	AB268019	986	AY794769	3514
Baloghia_inophylla	1331	AB233875	1203	AB233771	899	AY794707	3433
Bertya_sharpeana	1324	AB267935	1215	AB268039	0	-	2539
Blachia_siamensis	1324	AB267936	1203	AB268040	873	AY794727	3400
Caryodendron_orinocense	1325	AB233857	1203	AB233753	997	AY794760	3525
Chaetocarpus_africanus	1325	AB233858	1191	AB233754	854	AY794809	3370
Cheilosa_montana	1331	AB267910	1203	AB268014	0	-	2534
Chondrostylis_kunstleri	1331	AB233859	1203	AB233755	0	-	2534
Chrozophora_oblongifolia	1325	AB233860	1203	AB233756	0	-	2528
Claoxylon_glandulosum	0	-	1209	LK021406	998	HG971843	2207
Clutia_augustifolia	1321	AB233861	1191	AB233757	0	-	2512
Cnidoscolus_aconitifolius	1325	AB267937	1203	AB268041	946	MH935283	3474
Cocconerion_minus	1323	AB267938	1203	AB268042	0	-	2526
Codiaeum_peltatum	1331	AB233876	1203	AB233772	0	-	2534
Croton_insularis	1331	AB233877	1203	AB233773	697	AY971308	3231
Crotonogynopsis_usambarica	1331	AB233862	1209	AB233758	1008	HG971853	3548
Dalechampia_spathulata	1331	AB233863	1188	AB233759	1080	Y794754	3599
Discoglypremna_caloneura	1324	AB267916	1209	AB268020	1095	AY794802	3628
Ditaxis_montevidensis	1324	AB233865	1203	AB233761	0	-	2527
Ditta_myricoides	1311	AB267939	1212	AB268043	1006	AY794675	3529
Droceloncia_rigidifolia	1318	AB267917	1203	AB268021	0	-	2521
Drypetes_littoralis	1331	AB233926	1203	AB233822	0	-	2534
Elateriospermum_tapos	1322	AB267940	1203	AB268044	964	AY794678	3489
Endospermum_moluccanum	1331	AB267941	1206	AB268045	976	AY794671	3513
Erythrococca_anomala	1324	AB267918	1215	AB268022	0	-	2539
Euphorbia_acuta	1300	JN249261	1203	HQ645674	982	JN249596	3485
Euphorbia_angusta	0	-	1203	HQ645678	381	HQ645528	1584
Euphorbia_atoto	1331	AB267955	1203	AB268059	402	HQ645536	2936

	rbcL		matK		trnL		
**Species**	**Length (bp)**	**Accession**	**Length (bp)**	**Accession**	**Length (bp)**	**Accession**	**Total (bp)**
Euphorbia_atrococca	0	-	0	-	1031	MH490365	1031
Euphorbia_celastroides	1106	JN249280	1203	HQ645698	1053	JN249615	3362
Euphorbia_grantii	1331	AB233888	1200	AB233784	1032	JN249654	3563
Euphorbia_haeleeleana	0	-	1221	KC019490	0	-	1221
Euphorbia_herbstii	0	-	0	-	1036	MH490446	1036
Euphorbia_humifusa	1331	AB233884	1203	AB233780	406	HQ645587	2940
Euphorbia_johnstonii	0	-	1203	HQ645742	386	HQ645592	1589
Euphorbia_kuwaleana	0	-	1203	HQ645743	1048	MH490361	2251
Euphorbia_leucocephala	1300	JN249340	637	GU214862	978	JN249672	2915
Euphorbia_neocrispa	1324	AB267958	1224	AB268062	0	-	2548
Euphorbia_olowaluana	0	-	1203	HQ645766	1042	MH490363	2245
Euphorbia_rockii	0	-	1004	HQ645791	1022	MH490489	2026
Euphorbia_tithymaloides	1331	AB267959	1203	AB268063	0	-	2534
Excoecaria_cochinchinensis	1331	AB233885	1203	AB233781	959	AY794619	3493
Fontainea_venosa	1324	AB233878	1203	AB233774	872	AY794708	3399
Glycydendron_amazonicum	1324	AB267942	1209	AB268046	964	AY794681	3497
Hevea_brasiliensis	1316	NC_15308	1197	NC_15308	988	NC_15308	3501
Hippomane_mancinella	1324	AB267956	1203	AB268060	983	AY794616	3510
Homalanthus_nutans	1331	AB267957	1203	AB268061	961	JN249590	3495
Homalanthus_populifolius	572	KM392269	757	KM392242	0	-	1329
Humiria_balsamifera	1324	AB233889	1197	AB233785	0	-	2521
Hura_crepitans	1331	AB233886	1203	AB233782	984	AY794636	3518
Irvingia_malayana	1331	AB233892	1195	AB233788	0	-	2526
Jatropha_integerrima	1331	AB233879	1215	AB233775	1034	AY794685	3580
Jatropha_multifida	687	KP898357	749	LC461834	937	KP868720	2373
Jatropha_curcas	1315	NC_12224	1212	NC_12224	1024	NC_12224	3551
Klaineanthus_gaboniae	1331	AB267944	1203	AB268048	887	AY794668	3421
Lasiocroton_macrophyllus	1324	AB267919	1203	AB268023	0	-	2527
Lasiocroton_microphyllus	1320	AB267920	1203	AB268024	990	HG971893	3513
Lobanilia_bakeriana	1324	AB267921	1209	AB268025	1022	AY794779	3555
Macaranga_aleuritoides	1331	AB267922	1203	AB268026	988	Q899191	3522
Macaranga_tanarius	1331	MW297079	1203	MW297079	994	MW297079	3528
Mallotus_japonicus	1309	MW244068	1203	MW244068	990	MW244068	3502
Manihot_carthagenensis	1331	MK430314.1	0	-	964	EU518901	2295
Manihot_esculenta	1313	NC_10433	1209	NC_10433	1000	NC_10433	3522
Mareya_micrantha	1324	AB267924	1197	AB268028	1026	AY794774	3547
Melanolepis_multiglandulosa	1331	AB267925	1203	AB268029	988	AY794733	3522
Mercurialis_leiocarpa	1331	AB233867	1203	AB233763	0	-	2534
Micrandra_spruceana	1323	AB267945	1212	AB268049	0	-	2535

	rbcL		matK		trnL		
**Species**	**Length (bp)**	**Accession**	**Length (bp)**	**Accession**	**Length (bp)**	**Accession**	**Total (bp)**
Micrandropsis_scleroxylon	1321	AB267946	1206	AB268050	0	-	2527
Necepsia_afzelii	1324	AB233868	1203	AB233764	986	DQ991514	3513
Neoboutonia_mannii	1324	AB233881	1215	AB233777	889	AY794723	3428
Neoscortechinia_forbesii	1322	AB267911	1197	AB268015	0	-	2519
Neoscortechinia_kingii	1322	AB267912	1203	AB268016	971	AY794806	3496
Ophellantha_spinosa	1324	AB267947	1209	AB268051	685	AY971344	3218
Ostodes_paniculata	1322	AB267948	1215	AB268052	881	AY794725	3418
Paranecepsia_alchorneifolia	1315	AB267926	1203	AB268030	0	-	2518
Philyra_brasiliensis	1324	AB267927	1209	AB268031	1001	AY794740	3534
Pimelodendron_griffithianum	1331	AB233887	1187	AB233783	0	-	2518
Plukenetia_polyadenia	1324	AB267928	1194	AB268032	0	-	2518
Pycnocoma_cornuta	1324	AB233870	1203	AB233766	0	-	2527
Ricinocarpos_pinifolius	1324	AB267949	1212	AB268053	0	-	2536
Ricinus_communis	1331	NC_16736	1203	NC_16736	981	NC_16736	3515
Romanoa_tamnoides	1320	AB233872	1194	AB233768	1037	Y794757	3551
Sacoglottis_sp.	1323	AB233890	1197	AB233786	0	-	2520
Schinziophyton_rautanenii	1324	AB233882	1215	AB233778	0	-	2539
Seidelia_triandra	1324	AB267929	1203	AB268033	983	AY794762	3510
Speranskia_cantonensis	1326	AB267930	1203	AB268034	1025	AY794735	3554
Spirostachys_africana	1324	AB267960	1203	AB268064	977	Y794621	3504
Sumbaviopsis_albicans	1324	AB267931	1209	AB268035	986	Y794732	3519
Suregada_aequorea	1331	AB267950	1203	AB268054	0	-	2534
Suregada_glomerulata	1331	AB267951	1203	AB268055	0	-	2534
Tetrorchidium_rubrivenium	1315	AB267952	1212	AB268056	0	-	2527
Tragia_betonicifolia	1324	AB267932	1194	AB268036	0	-	2518
Tragiella_anomala	1324	AB267933	1194	AB268037	1032	AY794753	3550
Triadica_sebifera	1331	AB267961	1209	AB268065	1021	Y794649	3561
Trigonopleura_malayana	1331	AB233873	1194	AB233769	0	-	2525
Vernicia_montana	1316	MW297080.1	1215	MW297080.1	1049	MW297080	3580
Vernicia_fordii	1301	KY628420	0	-	0	-	1301
Wetria_insignis	1324	AB267934	1203	AB268038	0	-	2527

***Table S3.* Environmental variables correlated to PCA axes (trees only)**

	Correlation Coefficient	*p*-value
**Dim 1**		
Temperature of the Wettest Quarter	0.8690961	2.699026e-83
Minimum Temperature of the Coldest Month	0.8645576	1.826855e-81
Vegetation Cover	0.7552094	1.035758e-50
Precipitation of the Driest Month	0.3539213	2.508441e-09
Climatic Water Deficit	0.2165732	3.551292e-04
Precipitation Seasonality	-0.2360332	9.570400e-05
Solar Radiation	-0.7002899	7.997929e-41
**Dim 2**		
Precipitation Seasonality	0.7008964	6.400334e-41
Climatic Water Deficit	0.6665530	8.467696e-36
Minimum Temperature of the Coldest Month	0.4632755	1.155631e-15
Temperature of the Wettest Quarter	0.4485016	1.144784e-14
Solar Radiation	0.4037758	6.238385e-12
Vegetation Cover	-0.2841251	2.274431e-06
Precipitation of the Driest Month	-0.7682722	1.856038e-53
**Dim 3**		
Climatic Water Deficit	0.6439586	8.730307e-33
Precipitation of the Driest Month	0.2994582	5.898452e-07
Solar Radiation	0.2484549	3.904704e-05
Vegetation Cover	-0.2703221	7.171872e-06
Precipitation Seasonality	-0.5406903	9.516836e-22

***Table S4.* Environmental variables correlated to PCA axes (all lifeforms)**

	Correlation Coefficient	*p*-value
**Dim 1**		
Minimum Temperature of the Coldest Month	0.85293675	2.680179e-180
Temperature of the Wettest Quarter	0.83826172	2.393567e-168
Climatic Water Deficit	0.68588944	3.621171e-89
Precipitation Seasonality	0.54686588	1.186145e-50
Solar Radiation	0.09338178	1.877688e-02
Precipitation of the Driest Month	-0.56734656	3.336257e-55
**Dim 2**		
Vegetation Cover	0.8112428	3.399612e-149
Precipitation of the Driest Month	0.5943777	1.014873e-61
Temperature of the Wettest Quarter	0.5000729	2.339880e-41
Minimum Temperature of the Coldest Month	0.4872993	4.629512e-39
Climatic Water Deficit	-0.1930343	9.924023e-07
Precipitation Seasonality	-0.4538317	1.745728e-33
Solar Radiation	-0.8088022	1.283092e-147
**Dim 3**		
Climatic Water Deficit	0.6144706	5.582018e-67
Precipitation of the Driest Month	0.3464571	2.731684e-19
Solar Radiation	0.1234679	1.857199e-03
Vegetation Cover	-0.3262061	3.687601e-17
Precipitation Seasonality	-0.5495276	3.165038e-51
**Dim 4**		
Solar Radiation	0.51276391	9.761776e-44
Vegetation Cover	0.35460097	3.425228e-20
Climatic Water Deficit	-0.09449286	1.740606e-02
Precipitation of the Driest Month	-0.14115937	3.675769e-04
Precipitation Seasonality	-0.28336754	3.738228e-13

***Table S5.* Soil variables correlated to PCA axes (trees only)**

	Correlation Coefficient	*p*-value
**Dim 1**		
Organic Matter	0.8703556	2.11E-51
Water Permeability	0.6360706	7.30E-20
Shrink-Swell Potential	-0.4565908	9.02E-10
pH	-0.6425377	2.35E-20
**Dim 2**		
Cation Exchange Capacity	0.8675693	1.04E-50
Water Permeability	0.5182415	1.39E-12
pH	0.5162413	1.75E-12
Shrink-Swell Potential	0.418833	2.63E-08
Organic Matter	0.1848592	1.82E-02
**Dim 3**		
Shrink-Swell Potential	0.7374708	3.06E-29
Organic Matter	0.2214541	4.50E-03
Water Permeability	-0.2365187	2.37E-03
pH	-0.4576132	8.18E-10
**Dim 4**		
Water Permeability	0.494389	1.98E-11
Shrink-Swell Potential	0.2666742	5.80E-04
Cation Exchange Capacity	-0.4585921	7.45E-10
